# Transient Aggregation-Prone States in Disordered Proteins as Therapeutic Targets: The Amyloid‑β Case

**DOI:** 10.1021/acs.jcim.6c00270

**Published:** 2026-04-15

**Authors:** Margherita Bini, Valentina Tozzini, Luca Bellucci

**Affiliations:** † Scuola Normale Superiore, P.za S. Silvestro 12, 56127 Pisa, Italy; ‡ Istituto Nanoscienze, Consiglio Nazionale delle Ricerche (CNR-NANO), P.za S. Silvestro 12, 56127 Pisa, Italy; § INFN, Sezione di Pisa, Largo Bruno Pontecorvo, 56127 Pisa, Italy

## Abstract

The amyloid-β (Aβ) peptide is an intrinsically disordered protein whose self-association into toxic oligomers underlies Alzheimer’s disease. Because of its dynamic and heterogeneous nature, identifying the conformational states that nucleate aggregation remains a central challenge. In this work, we introduce a chemically interpretable descriptor of amyloidogenic propensity derived from self-docking analyses of conformational ensembles generated through temperature-replica exchange molecular dynamics (T-REMD) using different and complementary force fields. This descriptor classifies individual conformers within the generated ensembles according to their intrinsic aggregation tendency, enabling the identification of metastable, aggregation-prone states. The resulting ensembles reproduce experimental observables, and their classification based on amyloidogenic propensity provides a consistent structural basis for the rationalization and study of these metastable conformers. As a test, we demonstrate that the molecular chaperone DNAJB6, experimentally known to bind amyloidogenic conformations, preferentially interacts with aggregation-prone conformers, thus supporting both the proposed protocol and the consistency of the classification scheme. More broadly, this framework outlines a potentially generalizable strategy to identify metastable states in intrinsically disordered proteins as prospective pharmacological targets to help develop drugs or biomolecules capable of inhibiting the early stages of their aggregation.

## Graphical abstract

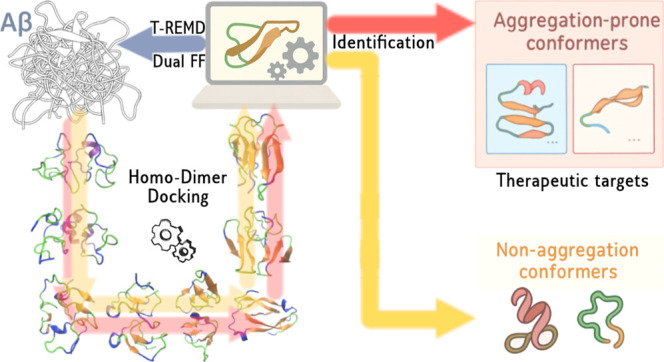

## Introduction

The pathogenesis of Alzheimer’s disease (AD), a neurodegenerative disorder marked by progressive cognitive decline and memory loss, involves multiple factors and mechanisms.^1^ One of these concerns the amyloidogenesis of the intrinsically disordered proteins amyloid-β (Aβ) peptides proceeding by the association of soluble monomers into oligomers, which evolve into insoluble fibrils with a β-sheet structure. Soluble oligomers are widely regarded as major contributors to neurotoxicity by altering neuronal function and synaptic plasticity, both essential for learning and memory, and by contributing to the amyloid cascade.^2^

As an intrinsically disordered protein (IDP), Aβ lacks a fixed three-dimensional structure and exists as a dynamic ensemble of interconverting conformations.^3^ Its energy landscape in solution comprises many closely related states, which may display different relative tendencies toward self-association.

Although the mechanism of Aβ self-assembly remains unclear, environmental factors such as pH, biomolecules, or nanoparticles can modulate this process.^4–7^ Antibodies^8,9^ and small molecules^10–15^ also interfere, either stabilizing nonamyloidogenic conformers or preferentially interacting with aggregation-prone states.

Computational modeling is essential to complement experiments by describing Aβ dynamics in solution, but its effective use requires statistically robust sampling of the conformational ensemble and reliable, operational evaluation of the amyloidogenic propensity of sampled states. While classical Molecular Dynamics (MD) combined to Temperature Replica Exchange MD (T-REMD)^16^ is an effective approach to generate conformational ensembles,^17–19^ the evaluation of amyloidogenic propensity is more challenging.

A recurrent structural feature associated with aggregation is the β-hairpin motif between hydrophobic residues 17–21 and the C-terminal region beyond residue 29, which promotes inter- or intramolecular interactions.^20,21^ This motif has been validated both experimentally, using sequence variants that stabilize or disrupt it,^20,22^ and computationally, through simulations started from experimental or AlphaFold-predicted structures.^20^ Beyond this motif, aggregation-prone conformations are often associated with intermediate states between globular and fibrillar forms, typically within given limited ranges of radius of gyration (*R*_g_) and solvent-accessible surface area (SASA).^13,23^ Although informative, these global descriptors lack robustness and specificity, highlighting the need for more reliable indicators.

Our work addresses this gap by generating a large data set of Aβ conformations and classifying them as aggregation-prone or nonprone within a descriptor-based, ensemble-relative framework. We focus our simulation on the more aggregation-prone isoform Aβ1–42 (Aβ42), strongly implicated in early pathology.^2^ We performed two independent T-REMD simulation series with the force fields (FFs) a99SB-UCB^24,25^ and OPLS,^26^ for a cumulative simulation time of ∼150 μs. This dual-FF strategy enabled extensive sampling of the conformational landscape and allowed us to assess the robustness of the results with respect to FF parametrization. To classify conformations, we developed a descriptor inspired by Bosio et al.,^13^ obtained by docking each conformation against a copy of itself and using the docking score as a proxy for self-association propensity. Unlike traditional structural metrics, this interaction-based ensemble-relative descriptor defines amyloidogenicity as an emergent property of conformational self-recognition rather than that of any single structure.

Although most previous studies focused on mature fibrils, the critical pathological transition in AD involves the conversion of nontoxic ensembles into transient amyloidogenic intermediates, which represent interesting therapeutic targets. Identifying and characterizing them are essential to understand the earliest steps of amyloidogenesis and to develop targeted strategies. Within this framework, our objective is to build a large data set of Aβ conformations, systematically classified into aggregation-prone and nonprone, as a reference for drug discovery workflows. Specifically, our objective is to provide a structurally informed and pharmacologically relevant set of aggregation-prone Aβ conformations suitable for structure-based virtual screening.

The resulting data set is also tested against DNAJB6, a member of the Hsp40 family shown to suppress early Aβ nucleation by engaging aggregation-prone species;^27^ in our framework, this provides an operational benchmark for conformer-selective recognition. While the precise oligomerization state of the species recognized by DNAJB6 in solution remains difficult to resolve experimentally, docking against monomeric conformers provides a controlled way to probe conformer-selective signatures that are likely to be enriched in early intermediates. A key strength of our approach is the ability to distinguish aggregation-prone from nonprone Aβ conformations, enabling docking against both subsets to assess preferential binding and to benchmark the proposed classification strategy.

## Results and Discussion

The overall workflow of the study is summarized in Figure 1. Broadly sampled conformational ensembles of Aβ42 monomers were generated through two independent T-REMD simulation series using the OPLS and a99SB-UCB FFs (Figure 1a). We analyzed conformations from the first 10 replicas of each FF, spanning temperatures from 300 to 318 K, corresponding to a biologically relevant range from ambient to near-physiological conditions. For computationally demanding analyses, we evaluated only the last 200 ns of the trajectories after verifying consistency with the preceding 200 ns block to assess the robustness of the results. These ensembles were characterized by using standard structural descriptors and NMR observables. Representative conformations obtained after clustering were evaluated for aggregation propensity through homodimer docking using the protein–protein HADDOCK engine.^28^ The HADDOCK score (HS) of the resultant complexes was used as a quantitative descriptor to classify conformations into aggregation-prone or nonprone states within the present computational framework (Figure 1b).

**Figure 1:**
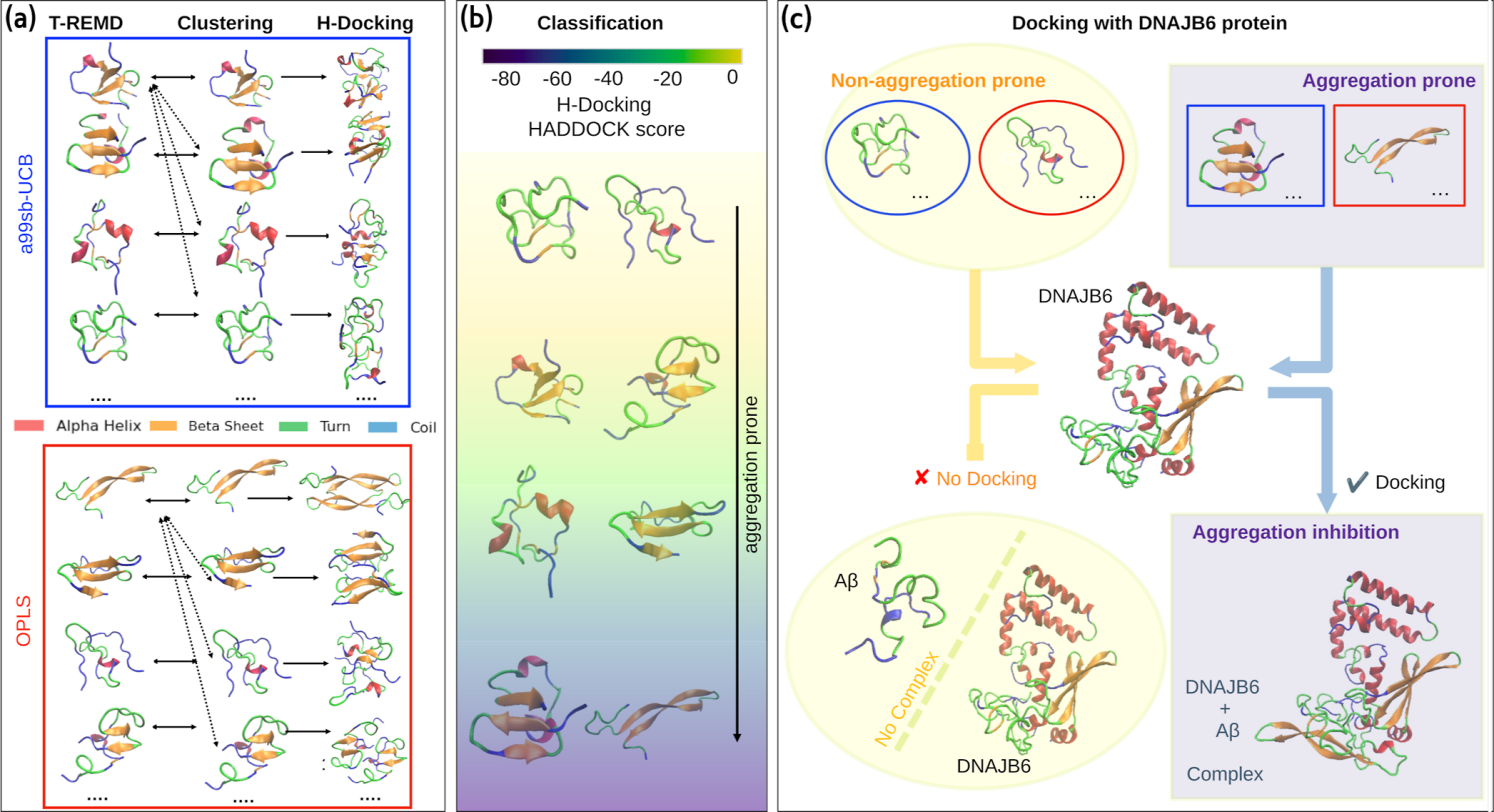
Multistage computational workflow to identify the aggregation propensity of Aβ conformations: (a) T-REMD sampling with dual FFs, followed by characterization, clustering, and homodimer docking of each representative cluster conformation; (b) evaluation of aggregation propensity of each representative conformation exploiting HS of the homodimer complex; and (c) validation through docking against the molecular chaperone DNAJB6.

This classification was further examined by testing the binding of DNAJB6, which selectively recognizes aggregation-prone states of Aβ, thereby providing a biologically motivated benchmark (Figure 1c). The structural models and sequences for both proteins are shown in Figure S1.

Details are reported in the Methods and Computational Details section.

### Intrinsically Disordered Nature of Aβ42 from T-REMD: Experimentally Consistent Ensembles

Our analysis reveals that both FFs consistently reproduce the intrinsically disordered nature of Aβ42, in agreement with previous studies.^4,29,30^ For each simulation, convergence is confirmed by the substantial overlap of structural descriptor distributions (*R*_g_, *R*_ee_, SASA, and secondary structure) across different T-REMD time blocks (see Figures S3 and S4).

The average *R*_g_ is 1.03 nm for OPLS and 1.05 nm for a99SB-UCB, both in agreement with the experimental value of 1.2 ± 0.2 nm (Table 1). The *R*_g_ distribution shows two regions: a dominant peak at ∼1 nm, corresponding to compact conformations, and a tail up to ∼1.5 nm, associated with elongated states (Figure 2a). Consistently, the end-to-end distance (*R*_ee_) distribution (Figure S5) displays a small peak at short distances and a broader peak around ∼2 nm for both FFs. The average *R*_ee_ agrees with other MD-derived values^29^ (Table 1), although it is slightly below experimental estimates^31^ as commonly observed in MD simulations (discussion in the Supporting Information, Figure S5). While the mean *R*_g_ values are comparable for the two FFs, the distributions indicate that a99SB-UCB favors more extended conformations, as evidenced by the *R*_g_ tail near ∼1.4 nm (Figure 2a, inset) and the broader *R*_ee_ distribution extending beyond 3 nm (Figure S5).

**Table 1:** Simulated Properties of Aβ42 from the First 10 T-REMD Replicas for OPLS, a99SB-UCB, and Their Combined Ensemble, Compared with Experimental Values

quantity	OPLS	a99sb-UCB	OPLS + a99sb-UCB	exp./sim.
*R*_g_ (nm)	1.033 ± 0.007	1.047 ± 0.007	1.04 ± 0.007	1.19 ± 0.16 ^31^
*R*_ee_ (nm)	1.76 ± 0.01	1.88 ± 0.01	1.82 ± 0.01	4.3 ± 0.3 ^32^/2.6 ± 1 ^29^
α-helix (%)	0.9 ± 0.7	6 ± 2	3 ± 1	9.4 ^33^
β-sheet (%)	23 ± 3	15 ± 2	19 ± 3	25.2 ^33^
turn (%)	51 ± 2	48 ± 2	49.5 ± 2	65.4 ^33^
coil (%)	25 ± 2	32 ± 2	28.5 ± 2	-
SASA	33.9 ± 0.1	35.1 ± 0.1		44 ± 5 ^29^
β-sheet_(18–19–20)_ (%)	55.3	21.9	38.6	56.7 ^29^
χ^2^, *J*-coupling	4.24	3.46	3.23	-
RMSD, Cα-shift (ppm)	0.858	0.696	0.720	-

**Figure 2:**
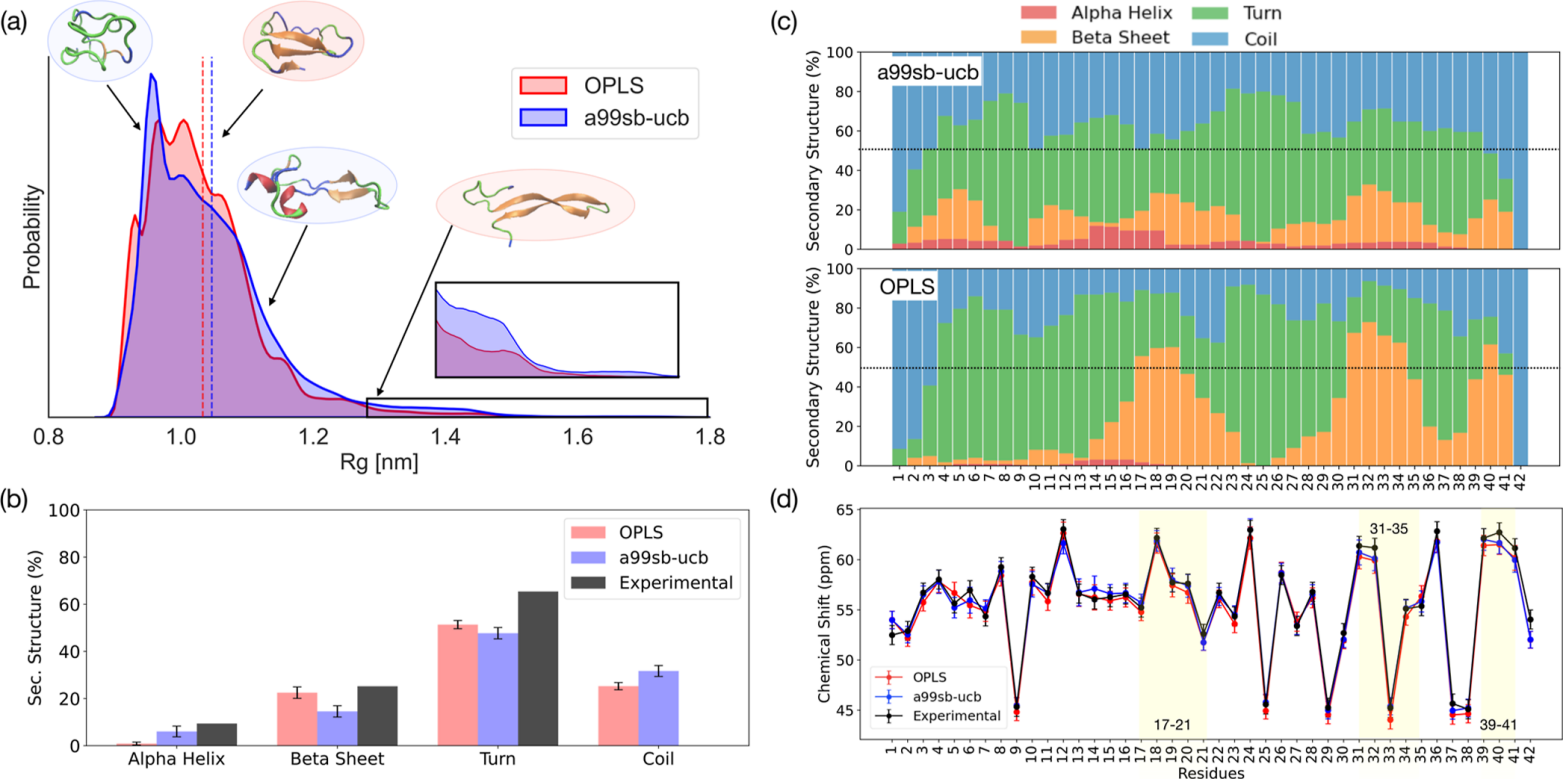
(a) Comparison of *R*_g_ distributions for the two FFs, averaged over the first 10 replicas of the full T-REMD. Dashed lines indicate mean values. Insets show representative globular and elongated structures for each FF; the right inset focuses on 1.3–1.8 nm, highlighting differences in the distribution tails. (b) Comparison of average secondary structure percentages over the first 10 full T-REMD replicas. Errors are computed with the block-average method. (c) Probabilities of secondary structure types (colors as indicated in the legend) for each residue, averaged over the last 200 ns of the first 10 replicas of the T-REMD simulations. (d) Comparison of experimental and computed residue chemical shifts, averaged over the last 200 ns of the first 10 T-REMD replicas. Regions where the two force fields differ in β-sheet content, and thus in chemical shifts, are highlighted in yellow.

The higher random-coil and lower β-sheet content predicted by a99SB-UCB compared to OPLS are consistent with these findings, as shown by the averaged secondary structure fractions compared in Figure 2b. OPLS markedly favors β-sheet conformations, yielding a percentage of approximately 23%, in close agreement with the experimental value reported for Aβ monomers^33^ and a very low percentage of α-helix (Table 1). In contrast, a99SB-UCB shows an enhanced propensity for helices, ∼6%, close to the experimental value of 9.4% but considerably lower β-sheet percentage (Table 1). This complementarity between the two FFs suggests that combining the conformational populations sampled by both provides a more balanced and experimentally consistent representation of the ensemble (see the fourth column of Table 1).

The average SASA values (33.9 nm^2^ for OPLS and 35.1 nm^2^ for a99SB-UCB, Figure S6) are consistent between the two simulations. The slightly larger SASA observed for a99SB-UCB reflects its higher population of disordered coil-like conformations, generally more extended and solvent-exposed, consistently with the secondary structure analysis. In addition, SASA exhibits a clear positive correlation with *R*_g_, a trend reported in our previous work^13^ (see Figure S7).

Figure 2c shows the residue-wise secondary structure content. In a99SB-UCB, the peptide predominantly adopts coil (blue) and turn (green) conformations with only minor α-helix (red) and β-sheet (orange) populations along the sequence. By contrast, OPLS predicts more β-sheets, especially in the central hydrophobic core (17–21) and the C-terminus (31–35, 39–41). The α-helix fraction is low in both FFs, though slightly higher in a99SB-UCB. Despite these quantitative differences, the two FFs display consistent secondary structure patterns, which also agree with previous simulations^18^ and NMR data.^34^ Specifically, the experimental *J*-couplings reported along the chain indicate that V18, F19, and F20 strongly favor β-strands,^35^ consistent with the extended conformations captured by OPLS (β-sheet_18–19–20_% in Table 1 and *J*-couplings in Figure S8). By contrast, a99SB-UCB yields a lower β-sheet fraction in this region (∼22%, Table 1), with random coil conformations dominating, suggesting that OPLS better captures the local β-propensity. In contrast to the β-sheet behavior, β-turns display comparable frequencies in both FFs: OPLS detects them in residues 4–16, whereas a99SB-UCB restricts them to residues 8 and 10 (green bars, Figure 2c). Both FFs agree on turns at 23–29 and 36–38. Random coils dominate the termini (1, 2, and 41–42) in both cases (light-blue bars, Figure 2c).

Experimentally, Roche et al.^35^ showed that Aβ42 monomers lack stable secondary structures being the ordered fraction below 50%. Consistently, our simulations keep residue-wise β-sheet propensities below this threshold, particularly in the case of a99SB-UCb, with OPLS slightly exceeding it for a small number of residues, supporting the intrinsically disordered nature of Aβ42 and its heterogeneous ensemble with dynamic β-sheet fluctuations.

The calculated chemical shifts (Figure 2d) and the ^3^ *J*_HN–Hα_ couplings (Figure S8) obtained with the two FFs were averaged over the last 200 ns of T-REMD and compared with the experimental data.^35^ To assess agreement, we used the RMSD for chemical shifts and the reduced χ^2^ for *J*-couplings^30^ (see Methods and Computational Details). Both FFs give results in reasonable agreement with the experiments (Table 1), with a99SB-UCB yielding a reduced χ^2^ of 3.46 and an RMSD of 0.696, outperforming OPLS (χ^2^ = 4.24, RMSD = 0.858). These values compare favorably with previous simulations.^29,30^

On average, OPLS shifts are lower than those of a99SB-UCB (Figure 2d), consistent with its higher β-sheet content, as β-sheets typically yield lower shifts than coils/turns and α-helices due to differences in hydrogen bonding and electronic environments. This effect is evident in the highlighted regions (yellow, Figure 2d). Combining the OPLS and a99SB-UCB ensembles gives an RMSD of 0.720 (Table 1), essentially averaging the two and slightly worsening the performance relative to that of a99SB-UCB alone.

*J*-couplings computed with a99SB-UCB are, on average, smaller than those from OPLS (Table S1 and Figure S8). This is consistent with the chemical shift results and with the higher β-sheet content predicted by OPLS, as extended conformations yield higher cos^2^(ϕ) values, thus larger *J*-couplings. When *J*-couplings are computed from the combined a99SB-UCB and OPLS conformations, χ^2^ decreases to 3.23, indicating partial error compensation, as also noted for the secondary structure.

Overall, both FFs reproduce experimental trends but with distinct biases: OPLS stabilizes β-sheets, sometimes excessively, while a99SB-UCB increases conformational diversity but under-stabilizes key β regions (e.g., 17–21). Together, they provide a more balanced description of Aβ in solution. This trend is reflected across multiple observables in the fourth column of Table 1, where values obtained from the combined a99SB-UCB and OPLS ensembles show improved overall consistency with experimental data compared with either force field alone.

### Aggregation-Prone Morphologies: An Ensemble-Docking Descriptor

Representative cluster structures from the last simulation block were evaluated for their ability to form stable homodimers using the docking score as an aggregation descriptor, capturing the emergent interaction-dependent nature of aggregation. The aggregation-propensity score obtained from HADDOCK homodimer docking classifies conformations as aggregation-prone (low score, HS ≤ −60) or nonaggregation-prone (high score, HS ≥ −20); see Figure 3 for representative aggregation-prone conformations and their resulting docking complexes, and Figure S9a for additional examples including both aggregation-prone and nonaggregation-prone conformations. Figures 4 and S10 show results of the analysis performed over the final 800–1000 ns interval, which are consistent with those from 600 to 800 ns (Figure S11), justifying the focus on the final interval.

**Figure 3:**
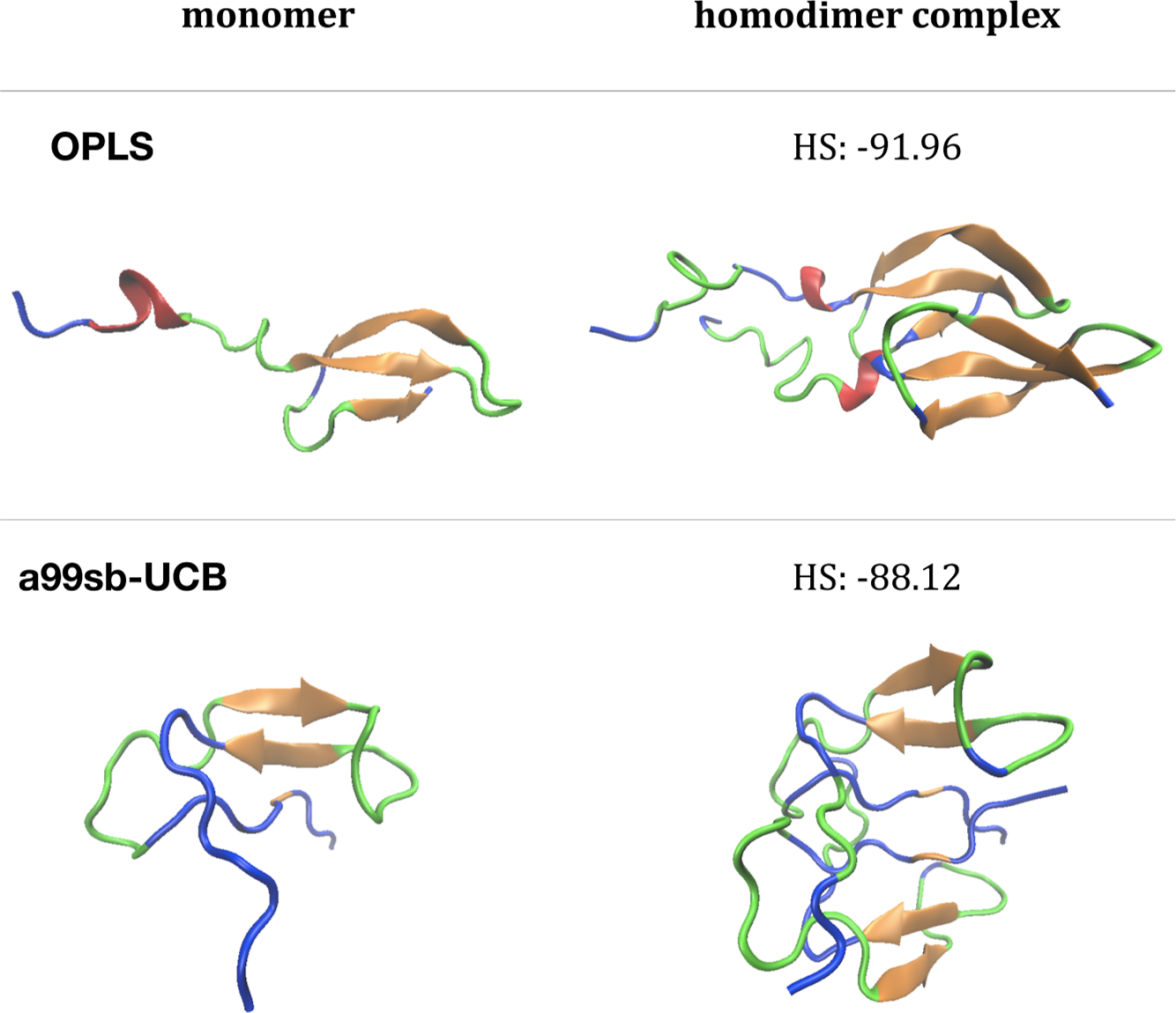
Example of aggregation-prone structures for the two FFs (left), the corresponding docked homodimer complexes (right), and their associated HS. The peptide is colored according to its secondary structure (blue: random coil; green: turn; orange: β-sheet; and red: α-helix).

**Figure 4:**
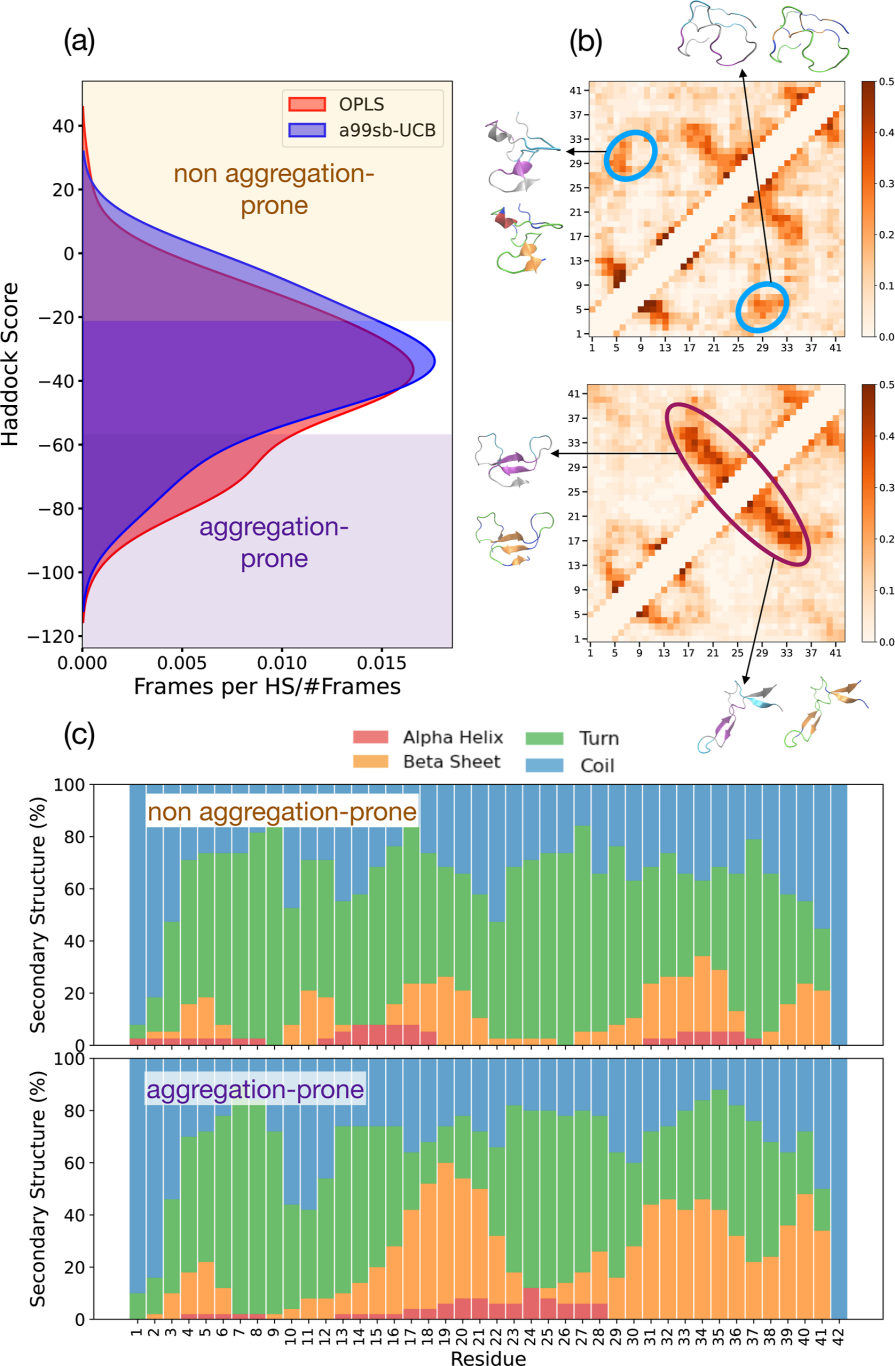
(a) HS value distributions for representative conformations, weighted by cluster populations, for the two FFs. (b) Intrapeptide interaction matrix for nonaggregation-prone (top) and aggregation-prone (bottom) conformations. Representative structures from each ensemble highlight the distinct interacting residues shown in the maps (cyan/purple ellipsoids). (c) Per-residue secondary structure distribution from low (top) and high (bottom) aggregation propensity structures. For this analysis, aggregation- and nonaggregation-prone ensembles from both FFs were merged to more comprehensively sample the conformational space.

Figure 4a shows the HS distribution for homodimer complexes derived from the representative structures of each cluster. The corresponding probability distribution indicates a higher population of aggregation-prone states for OPLS compared with a99SB-UCB (further details reported in Figure S10). Similar results were obtained for the 600–800 ns time window (Figure S11a).

Figure 4c shows the per-residue secondary structure distribution obtained by merging the ensembles from both FFs for the last block, providing a unified view of the structural features distinguishing the aggregation-prone and nonaggregation-prone states, consistent with the earlier time window (600–800 ns; Figure S11b).

The nonaggregation-prone ensemble (top panel) is dominated by *turn* and *coil* states (blue and green), forming a segmented pattern in which β-sheet elements (orange) are confined to residues 2–6, 10–13, 16–21, 30–35, and 38–41. These short β segments remain below 30% occupancy and isolated, while a small α-helical content (red) is observed around residues 13–18 and at both termini.

The aggregation-prone ensemble (bottom panel) exhibits an increase in β-sheet content with occupancies reaching or exceeding 50% in continuous β-sheet segments with a higher percentage at 17–21, 31–35, and 39–41, matching regions typically involved in amyloid formation as outlined in the Introduction and refined here. Comparison of the two panels indicates that the nonaggregation-prone states remain fragmented and turn-coil dominated, whereas the aggregation-prone states exhibit cooperative β-sheet formation centered on the hydrophobic core and C-terminal regions. The two FFs produce different conformations within each ensemble: OPLS tends to promote aggregation through a β-hairpin structure, whereas a99SB-UCB shows a similar increase in β-sheet content but also retains disordered regions around residues 10–12 and 29–30, which may modulate aggregation (see Figure S12 for more details on the importance of disordered regions).

Consistent with this mechanism, previous MD and experimental studies have shown that stabilizing the 17–21 β-hairpin and C-terminal region enhances aggregation, whereas disrupting them suppresses it.^20,22^ In addition, the importance of disordered regions is supported by previous MD simulations of the Aβ(25–35) peptide,^36^ which showed that β-hairpin structures dominate early aggregation, while flexible coil regions become more important later to recruit additional monomers.

Since the β-hairpin is defined by interactions between residues (17–21)–(31–35) and (31–35)–(39–41), its stabilization is confirmed in our study by the average intramolecular contacts of the aggregation-prone subset (purple in Figures 4b and S11c). Contacts between residues 1–10 and 25–35 (cyan in Figures 4b and S11d) occur specifically in nonaggregation-prone conformations and are largely absent in aggregation-prone states. These interactions increase the separation between residues 17–21 and 31–35, preventing formation of the β-hairpin (see representative structures in Figure 4b).

To examine whether these intramolecular features are reflected in intermolecular interactions, we analyzed the dimers generated by homodimer docking (Figure S9a). The interaction probability matrices (Figure S9b) reveal preferred contacts involving residues 1–8, 16–24, 28–36, and 37–42, consistent with the β-structured segments identified in Figure 4c. These results support the role of the β-hairpin motifs in promoting aggregation. Moreover, comparison of dimers with low versus high HS (Figure S9, bottom row) shows that nonaggregation-prone conformations preferentially form turn-rich dimers, whereas aggregation-prone conformations give rise to dimers containing both parallel and antiparallel β-sheets, reminiscent of fibrillar assemblies (Figure S9c).

### Interaction-Based Descriptor versus Structural Metrics

The relationship between HS and secondary structure content, *R*_g_, and SASA was quantified for all representative conformations of the final temporal block (Figures 5 and S13) using Pearson correlation coefficients (eq 1 reported in the Methods and Computational Details section). These results are consistent with those obtained for the 600–800 ns interval (Figure S14 and Table S2).

**Figure 5:**
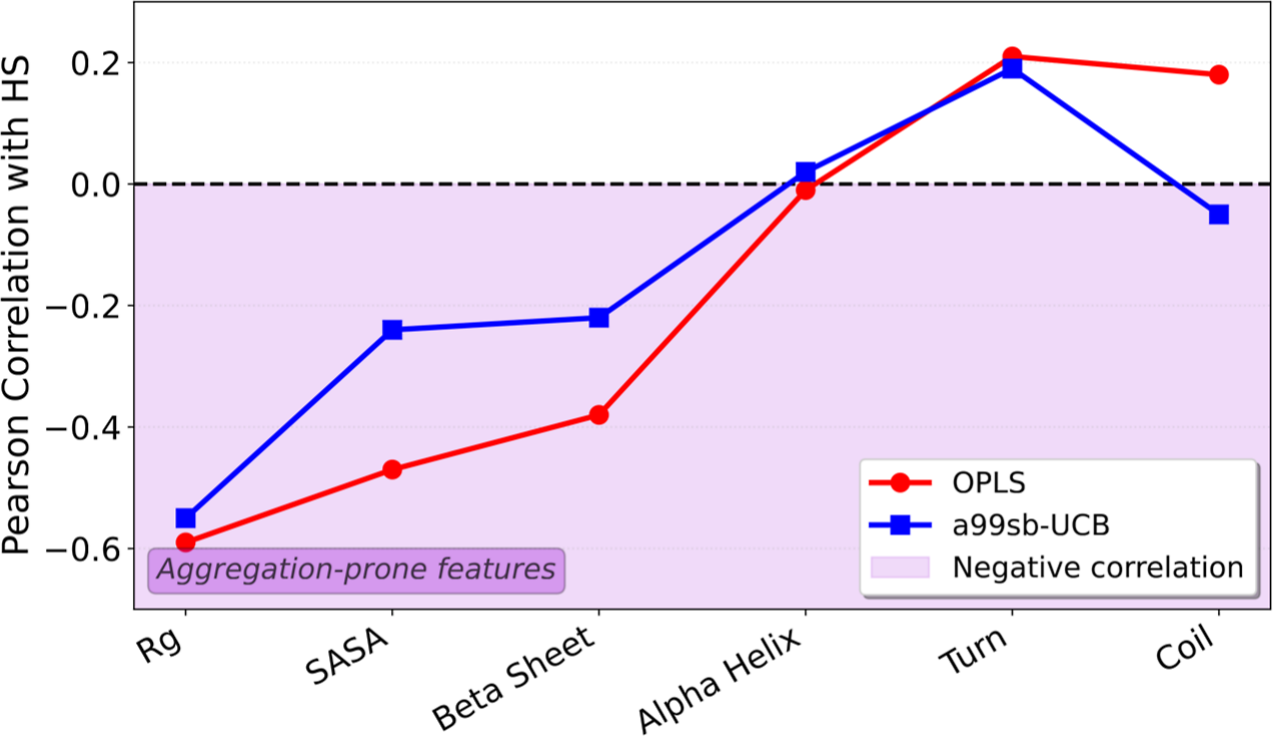
Pearson correlation coefficients between HS and structural metrics.

*R*_g_ and SASA display overall negative correlations with HS, more pronounced for *R*_g_ (see correlation plots in Figure S13b,c and in the preceding block, Figure S14c,d). Since more negative HS values correspond to higher aggregation propensity, a negative correlation here indicates that higher *R*_g_ or SASA is associated with an increased aggregation tendency. In the same way, OPLS displays a negative correlation between β-sheet content and HS (Figures 5, S13a, and S14a,b), similar to that observed for a99SB-UCB, although the substantial scatter indicates that this correlation is weak. The Pearson coefficient for the α-helical content is compatible with zero for both FFs (although the statistics are limited, especially for OPLS; Figure 2c), indicating essentially no correlation with the aggregation tendency. Conversely, the correlation between HS and turn content is positive and similar for both FFs, although slightly higher for OPLS. Interestingly, a99SB-UCB exhibits a negative correlation with coil content, suggesting that disordered regions in random-coil conformations may also contribute to aggregation, consistent with what we observed in the previous section. By contrast, OPLS shows a positive correlation, indicating that aggregation in this case is primarily associated with stabilization of β-sheet-like structures, whereas a99SB-UCB can also exploit coil-mediated interactions.

Overall, the Pearson correlation analysis shows that global structural metrics capture general trends with HS, but their relationships with HS display substantial scatter (Figures S13 and S14). This supports the view that such descriptors are informative but insufficiently robust or specific to assess the aggregation propensity of individual conformations, motivating the use of interaction-based descriptors as more direct indicators of self-association.

### DNAJB6 Supports Conformer-Selective Recognition

Figure 6 shows the docking results between DNAJB6 and both aggregation-prone and nonaggregation-prone Aβ conformations. Residues 132–221 of DNAJB6, spanning the linker and part of the C-terminal region, were used as the active site (see Methods and Computational Details and Figure S1b).

**Figure 6:**
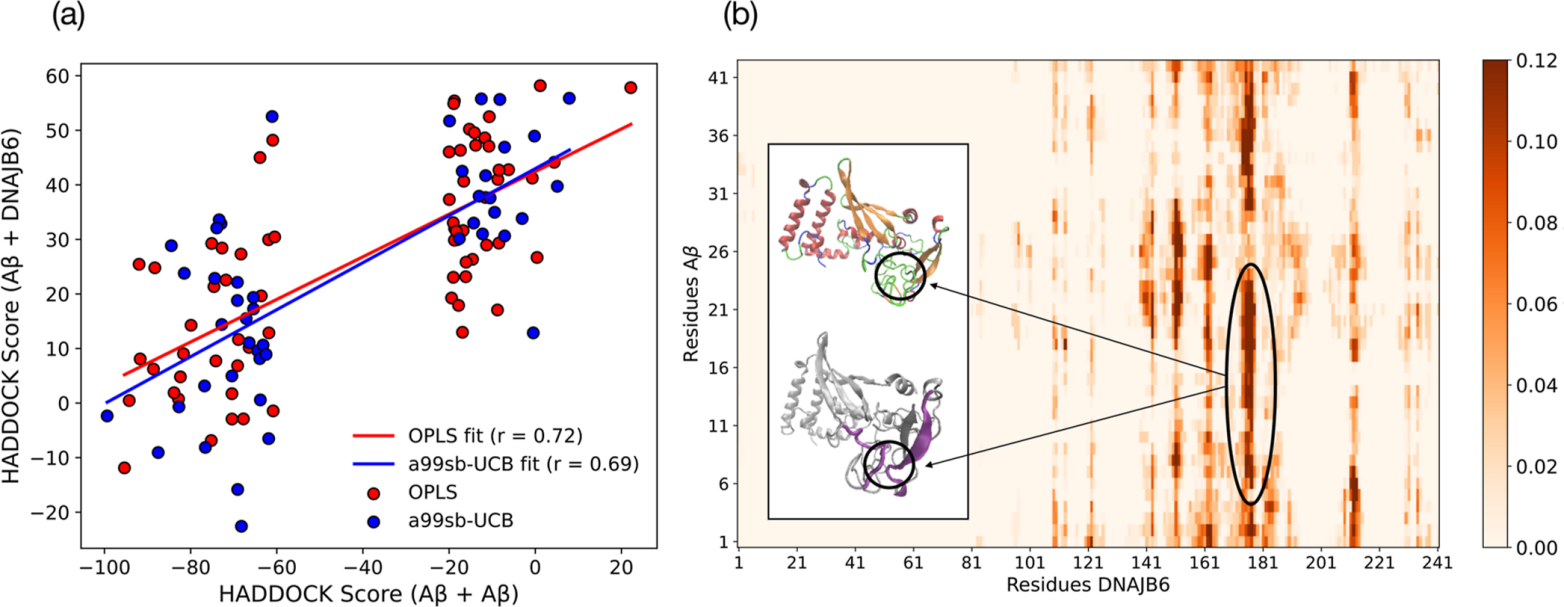
(a) Correlation between HS obtained from Aβ–Aβ docking and those obtained from Aβ–DNAJB6 docking for conformations obtained with OPLS and a99sb-UCB. (b) Interaction matrix of DNAJB6 + Aβ complexes.

The correlation between the Aβ–Aβ and Aβ-DNAJB6 HS values is reported in Figure 6a. A positive correlation is observed, with high Aβ + DNAJB6 HS corresponding to high Aβ + Aβ HS values, and similarly for low scores, indicating that DNAJB6 preferentially binds to aggregation-prone Aβ conformations. Across FFs, self-association propensity and DNAJB6 docking scores of different conformers are correlated, indicating a conformer-dependent interaction pattern consistent with DNAJB6’s aggregation-inhibitory role. This aligns with evidence that DNAJB6 suppresses Aβ aggregation by capturing aggregation-prone species, mainly at the prenucleation oligomeric stage.^27^ Accordingly, the aggregation-prone monomers identified here likely encode the structural signatures of the oligomers targeted by DNAJB6. Thus, docking against monomers offers a controlled way to probe conformer-selective features enriched in early aggregation intermediates rather than direct evidence of stable monomer binding in solution.

Figure 6b shows the interaction probability matrix between Aβ and DNAJB6. The highest interaction probability is in residues 172–180 of DNAJB6, rich in serine (S) and threonine (T). This region mainly contacts the hydrophobic segment 16–21 of Aβ (purple in Figure 6b), which we previously found to have high β-sheet content in aggregation-prone but not in nonaggregation-prone conformations (Figure 4b and c). The involvement of the S/T-rich region of DNAJB6 in the interaction is consistent with the finding that aggregation inhibition is lost when residues in the linker domain are mutated.^27^ This linker region is intrinsically amyloid-like and prone to self-assembly,^37^ but the presence of the N- and C-terminal domains in the intact protein prevents linker self-aggregation and maintains its availability for binding Aβ.^37^ The high affinity of this region for the β-hairpin structure observed in aggregation-prone Aβ conformations is consistent with the mechanism proposed in ref 27 where DNAJB6 is suggested to bind and stabilize such structures by forming hydrogen bonds between the hydroxyl groups of the S/T-rich region and the Aβ peptide, thereby targeting high-surface-energy β-sheet conformations.

Beyond serving as a biologically motivated benchmark, DNAJB6 shows that aggregation-prone conformers can be selectively recognized by their structural morphologies, providing a mechanistic basis for conformer-targeted inhibition. The ensembles generated here thus offer a foundation for ligand discovery and a framework to target metastable aggregation-nucleating conformers.

## Conclusion

In this work, we combined enhanced sampling MD (T-REMD) with two complementary FFs to generate ensembles of Aβ42 conformations, reducing parametrization bias and obtaining an experimentally consistent description of the intrinsically disordered peptide.

Representative conformations obtained from clustering were systematically evaluated through homodimer docking, yielding a physically grounded descriptor of the aggregation propensity. This approach reframes amyloidogenicity as an emergent, interaction-dependent property and highlights the morphological features of aggregation-prone conformers, including extended β-strands, solvent-exposed hydrophobic patches, and compact intramolecular contacts. The resulting data set provides a structurally characterized reference set of metastable conformers suitable for virtual screening and rational inhibitor design. Beyond Aβ42, the docking-based classification of aggregation-prone versus nonprone conformations represents a potentially generalizable protocol that can be readily extended to other amyloidogenic proteins, providing a transferable framework to investigate the structural determinants of early aggregation.

The biological relevance of this classification was further supported by DNAJB6, which preferentially recognizes aggregation-prone conformational signatures and is known to suppress early nucleation events. This observation illustrates how chaperones act on aggregation-competent conformational ensembles and supports the therapeutic value of targeting them.

Altogether, these findings provide mechanistic insight into the link between monomeric heterogeneity and pathogenic potential, reinforcing the central role of soluble-aggregation-prone conformers as key contributors to toxicity and privileged substrates for therapeutic intervention. DNAJB6 further illustrates this conformer-selective recognition. More generally, this shows that aggregation-competent conformers form a physically definable and targetable subensemble within the disordered state. In this perspective, our study consolidates the concept of morphological inhibitors^13^ as a generalizable strategy to uncover druggable metastable states in intrinsically disordered proteins, guiding the rational discovery of small molecules and biomolecular inhibitors that act at the earliest stages of protein misfolding.

## Methods and Computational Details

The molecular dynamics simulations of Aβ protein and subsequent analyzes were performed using GROMACS.2022.4 ^38^ and its associated tools.^39^ Molecular visualization was performed using VMD1.9.3.^40^ Protein–protein docking of Aβ-Aβ and Aβ-DNAJB6 protein was performed using HADDOCK2.4, a widely used and well-established platform to model complex biomolecular complexes.^28^

### Model System

The structures and sequences of the simulated proteins are shown in Figure S1. Aβ comprises 42 residues and can be divided into four regions (Figure S1a): a hydrophilic N-terminal segment, a hydrophobic core, a turn region, and a hydrophobic C-terminal tail. DNAJB6 contains two globular domains (Figure S1b)—the *J*-domain (JD) and the C-terminal domain (CTD)—connected by a flexible, serine/threonine/glycine/phenylalanine-rich linker. The monomeric structure used for docking simulations corresponds to that reported in ref 41.

### Ensemble Generation via T-REMD with Multiple FFs

Two independent T-REMD simulations were performed using two different FFs. In T-REMD, multiple replicas of the system are simulated in parallel at different temperatures, with periodic exchanges of configurations between replicas controlled by the Metropolis criterion.^16^ This technique improves sampling efficiency by facilitating the crossing of high-energy barriers, thus allowing for a more thorough exploration of the conformational landscape of the protein.

### Starting Structures for T-REMD

The initial structures for T-REMD simulations were selected from the conformational ensemble previously generated in ref 4 using the OPLS force field for the peptide and the TIP3P model for water, with the system solvated in a cubic box of 7.2 nm per side. Starting conformations were chosen to exhibit relatively large values of the radius of gyration (*R*_g_) and root-mean-square deviation (RMSD) from the average structure, computed as the mean of the representative cluster structures in ref 4 in order to enhance conformational diversity. These starting structures were used only to initialize independent replicas; the final ensembles were generated by extensive T-REMD sampling and do not retain the memory of the initial conformations. In total, 72 distinct conformations of Aβ42 were selected.

### Force Fields

Two different FFs, OPLS and a99SB-UCB, were used for two independent T-REMD simulations. The a99SB-UCB FF, which is an Amber-derived FF optimized for disordered proteins, was chosen for its superior agreement with experimental measurements of Aβ40.^30^ Instead, OPLS was used as a benchmark, given its different parametrization strategy based on quantum mechanical data and small molecules, and its tendency to favor β-sheet conformations compared to Amber-derived FFs,^42,43^ as well as for the good agreement between simulations and experimental data previously reported in ref 4. This behavior was also consistent with literature findings comparing different force fields on Aβ-derived systems.^43^ The solvent was modeled using the TIP3P water model.^44^

### T-REMD and MD Details

The starting conformations selected as described above were placed in a cubic periodic simulation box with a side length of 7.2 nm. This size was sufficient to prevent spurious interactions between the periodic images. It was consistent with experimental FRET estimates of the Aβ end-to-end distance, which peak around 4 nm and decay to zero at approximately 6 nm.^30,32^ Importantly, the box length also exceeded the maximal end-to-end distances observed in the present simulations and in previous atomistic studies,^4^ ensuring negligible self-interaction across periodic boundaries.

Each conformation was first equilibrated in the *NPT* ensemble (1 atm, 300 K) for 50 ns using the same force field that would be employed in the subsequent T-REMD simulation (a99SB-UCB and OPLS, respectively). The resulting structures exhibited high conformational variability (Figure S2) and are available in the repository as coordinate files for visualization (see the “Data and Software Availability” section). Subsequently, T-REMD simulations were carried out in the *NVT* ensemble using the average box size obtained from the preceding *NPT* runs. The replicas spanned a temperature range of 300–450 K, resulting in a total accumulated simulation time of 72 μs across 72 replicas for each force field, as illustrated in Figure 1a.

Simulations employed periodic boundary conditions (PBC) and an integration time step of 2 fs. Long-range electrostatic interactions were treated using the Particle Mesh Ewald (PME) method,^45^ with a grid spacing of 0.1 nm. Nonbonded interactions were computed with a 1.05 nm cutoff. All bond lengths were constrained using the LINCS algorithm.^46^ We used the stochastic velocity rescaling thermostat^47^ and the Parrinello–Rahman barostat^48^ to control temperature and pressure, respectively.

### Ensemble Validation Protocols

The conformational ensemble was validated by computing the radius of gyration (*R*_g_), end-to-end distance (*R*_ee_), chemical shifts, and *J* couplings and by comparing these quantities with experimental data. The experimental estimate of *R*_g_ was derived from the hydration radius (*R*_h_) measured by FRET-FCS experiments.^31^ All relations and computational details are reported in the Supporting Information, within a section entitled Ensemble Validation Protocols.

### Clustering and Aggregation-Prone Score Function

To account for the conformational heterogeneity of IDPs and to overcome the limitations of single-structure docking, an ensemble docking approach was employed.^49^ The procedure consists of generating an ensemble of representative conformations by clustering the trajectories of the first ten replicas, divided into 200 ns blocks, and selecting representative structures from clusters containing at least 100 members (Figure 1a,b). Clustering was performed using the GROMACS implementation of the “gromos” algorithm,^50^ considering only Cα atoms and applying a 0.28 nm Cα RMSD cutoff. The docking calculations were then performed with HADDOCK^28^ using the default protocol, which includes energy minimization, semiflexible refinement, and final refinement in explicit solvent. For each complex, HADDOCK provides a score that correlates with the binding free energy of complex formation.^28^ Structures with HS values obtained from homodimer docking were used as indicators of amyloidogenic propensity. The HS thresholds (defined in the Results section) were selected using a statistically based criterion, assuming an approximately Gaussian distribution and retaining conformers beyond ±1 standard deviation from the mean (upper and lower ∼16% tails).

Subsequently, both ensembles were structurally characterized. The secondary structure content and residue–residue contacts were analyzed by computing a probability interaction matrix, in which the probability of each residue pair being in contact was obtained by counting the fraction of frames in which their Cα atoms were within 8.2 Å. To further explore the relationship between structure and aggregation propensity, we quantified the correlation between HS and structural descriptors of aggregation, namely, the fractions of β-sheet, α-helix, coil, and turn. The Pearson correlation coefficient between HS and each descriptor *X* was calculated as:

1 $$r_{X}=\frac{\sum_{i=1}^{n}\left(X_{i}-\mathrm{X̅}\right)\left(HS_{i}-\overset{®}{HS}\right)}{\sqrt{\sum_{i=1}^{n}{\left(X_{i}-\mathrm{X̅}\right)}^{2}}\sqrt{\sum_{i=1}^{n}{\left(HS_{i}-\overset{®}{HS}\right)}^{2}}}$$

where X̅ and $\overset{®}{HS}$ denote the mean values of *X* and *HS*, respectively.

### Ensemble-Based Docking of Aggregation- and Nonaggregation-Prone Conformations with DNAJB6

Docking calculations were performed between representative Aβ monomers selected from the cluster analysis and the molecular chaperone DNAJB6. The DNAJB6 linker and CTD mediate client-protein recognition, with the conserved S/T-rich segment in the linker playing a critical role in antiamyloid activity. We therefore defined residues 132–221, spanning the S/T-rich linker and the CTD β-sheet, as active residues in the docking calculations. For each Aβ conformer, the HADDOCK score obtained from Aβ–DNAJB6 docking was compared with the corresponding Aβ–Aβ homodimer score to quantify the correlation between the self-association propensity and DNAJB6 recognition.

## Supplementary Material



## Data Availability

All data required to reproduce the computational workflow presented in this study are available on Zenodo (https://doi.org/10.5281/zenodo.18432004). This includes the Temperature Replica Exchange Molecular Dynamics (T-REMD) simulation input files, the HADDOCK protein–protein docking inputs and outputs, and the analysis files used to identify and characterize aggregation-prone conformational states of the Aβ42 peptide.

## Notes

Detailed computational procedures, additional figures and tables, together with the Ensemble Validation Protocols section and its subsections (PDF)
