# Supplementary material for: Transient Aggregation-Prone States in Disordered Proteins as Therapeutic Targets: The Amyloid‑β Case
Source: J Chem Inf Model. 2026 Apr 15;66(11):6591–601. doi: 10.1021/acs.jcim.6c00270 (PMC13250902; doi:10.1021/acs.jcim.6c00270)
Supplement: Supplementary file 1 [file ci6c00270_si_001.pdf]

Supporting Informations

Transient Aggregation-Prone States in Disordered Proteins as Therapeutic Targets: The Amyloid-β Case

Margherita Bini,[†,‡] Valentina Tozzini,[‡,†,¶] and Luca Bellucci[*,‡,†]

†*Scuola Normale Superiore, P.za S. Silvestro 12, 56127 Pisa, Italy*

‡*Istituto Nanoscienze, Consiglio Nazionale delle Ricerche (CNR-NANO), P.za S. Silvestro 12, 56127 Pisa, Italy*

¶*INFN, Sezione di Pisa, Largo Bruno Pontecorvo, 56127 Pisa, Italy*

E-mail: luca.bellucci@nano.cnr.it

Supporting Informations Overview

This Supporting Information provides additional analyses and validation supporting the main text. System setup, including Aβ conformations and the DNAJB6b docking model, is shown in Fig. S1. The structural characteristics of the initial conformation employed for T-REMD simulations are presented in Fig. S2. Convergence is assessed from the time evolution of structural descriptors (Fig. S3) and clustering (Fig. S4). Force fields are compared via R_{ee} (Fig. S5), SASA (Fig. S6), and their correlation (Fig. S7). Validation against experiment uses J-coupling constants (Table S1, Fig. S8). Aggregation propensity is characterized by docking and HADDOCK scores (Fig. S9, Fig. S10), block analysis with representative conformations (Fig. S11), and secondary-structure and contact analyses (Fig. S12). Correlations between aggregation propensity and structural descriptors are quantified via Pearson coefficients (Table S2) and correlation plots (Fig. S13, Fig. S14). The final section describes

ensemble-validation protocols, including calculation of structural and NMR observables and comparison with experimental data.

Figures and Tables

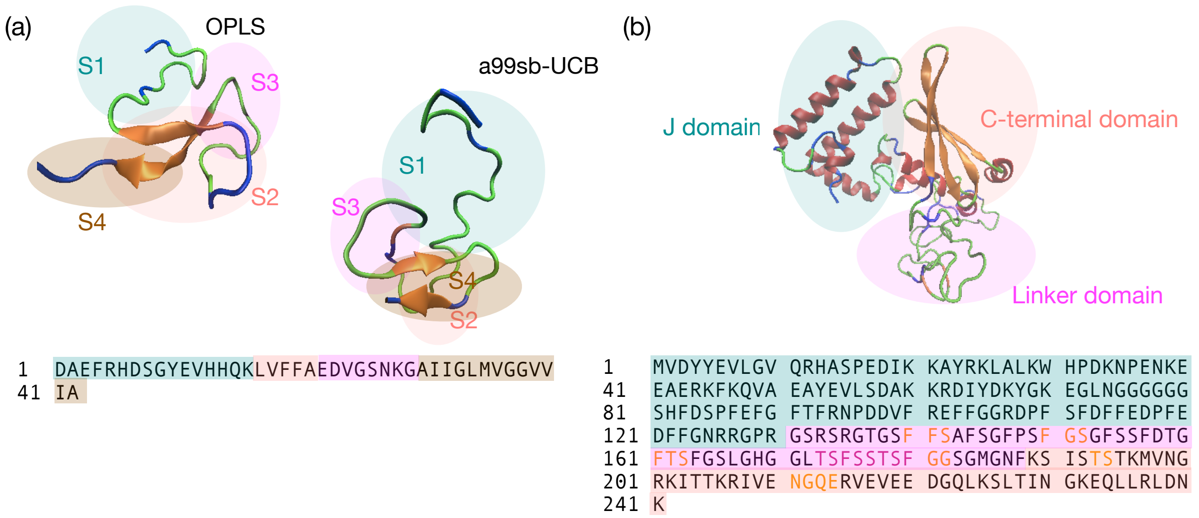

Figure S1: (a) Starting conformations of the lowest-temperature replicas from the T-REMD simulations of Aβ using the OPLS and a99SB-UCB force fields. The peptide is colored according to its secondary structure (blue: random coil; green: turn; orange: β-sheet; red: α-helix). The four sequence segments (S1–S4) are highlighted both in the structure and in the sequence. These segments differ in their physicochemical properties: S1 (residues 1–16) is mainly hydrophilic; S2 (residues 17–21) consists of hydrophobic and aromatic residues and corresponds to the “Aβ hydrophobic core”; S3 (residues 22–29) adopts a β-turn conformation in fibrils; and S4 (residues 30–42) forms the hydrophobic C-terminal tail. (b) Structural model of the DNAJB6b monomer used for docking with Aβ. The protein is colored according to its secondary structure (same color scheme as in (a)). In addition, the different regions are highlighted, with the J-domain shown in blue, the linker region in pink, and the C-terminal domain in orange.

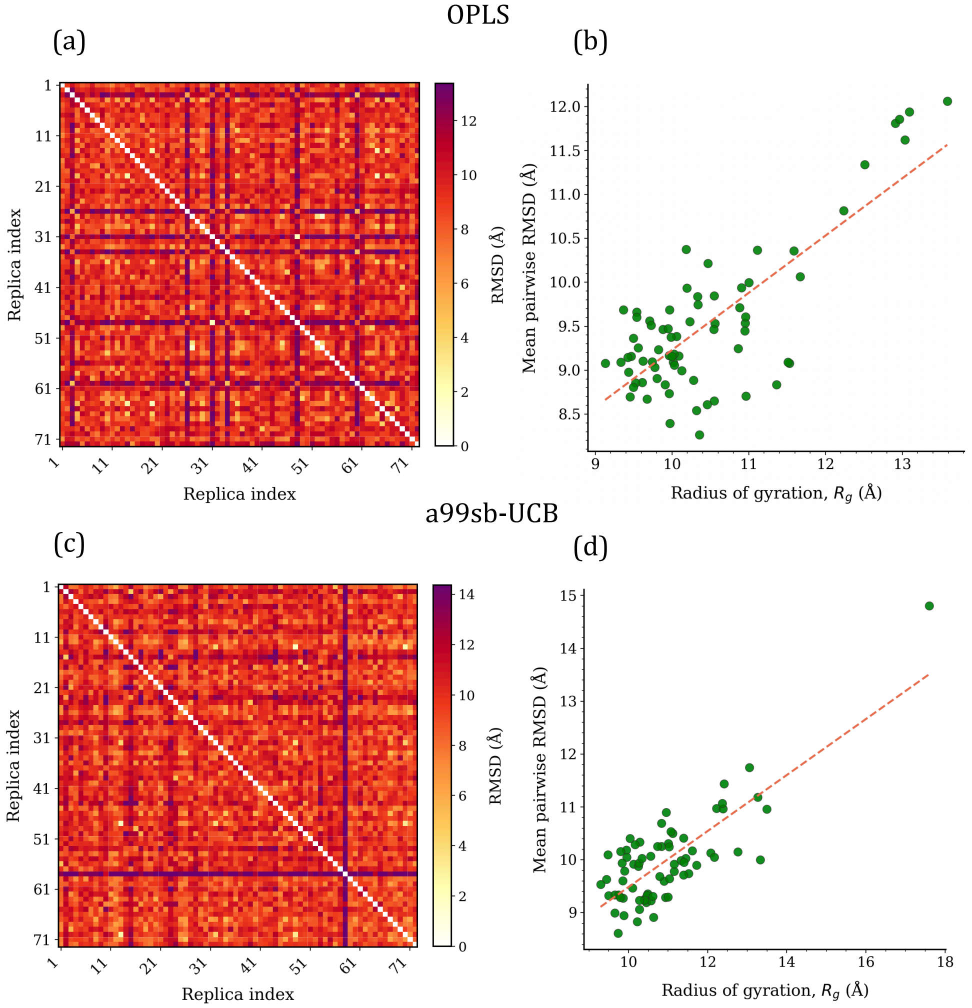

Figure S2: Structural diversity of the 72 Aβ42 starting conformations used in the T-REMD simulations. (a, c) Pairwise RMSD matrices computed over all 72 replica starting structures for the OPLS (a) and a99SB-UCB (c) ensembles, with RMSD values in Åindicated by the color scale. The predominantly red-colored off-diagonal entries reflect broad structural heterogeneity across the initial ensemble. (b, d) Correlation between the mean pairwise RMSD and the radius of gyration Rg for each starting conformation, for OPLS (b) and a99SB-UCB (d). The positive correlation (dashed line) confirms that more extended conformations tend to be more structurally distinct from the rest of the ensemble, consistent with a broad sampling of both compact and elongated Aβ42 monomeric states.

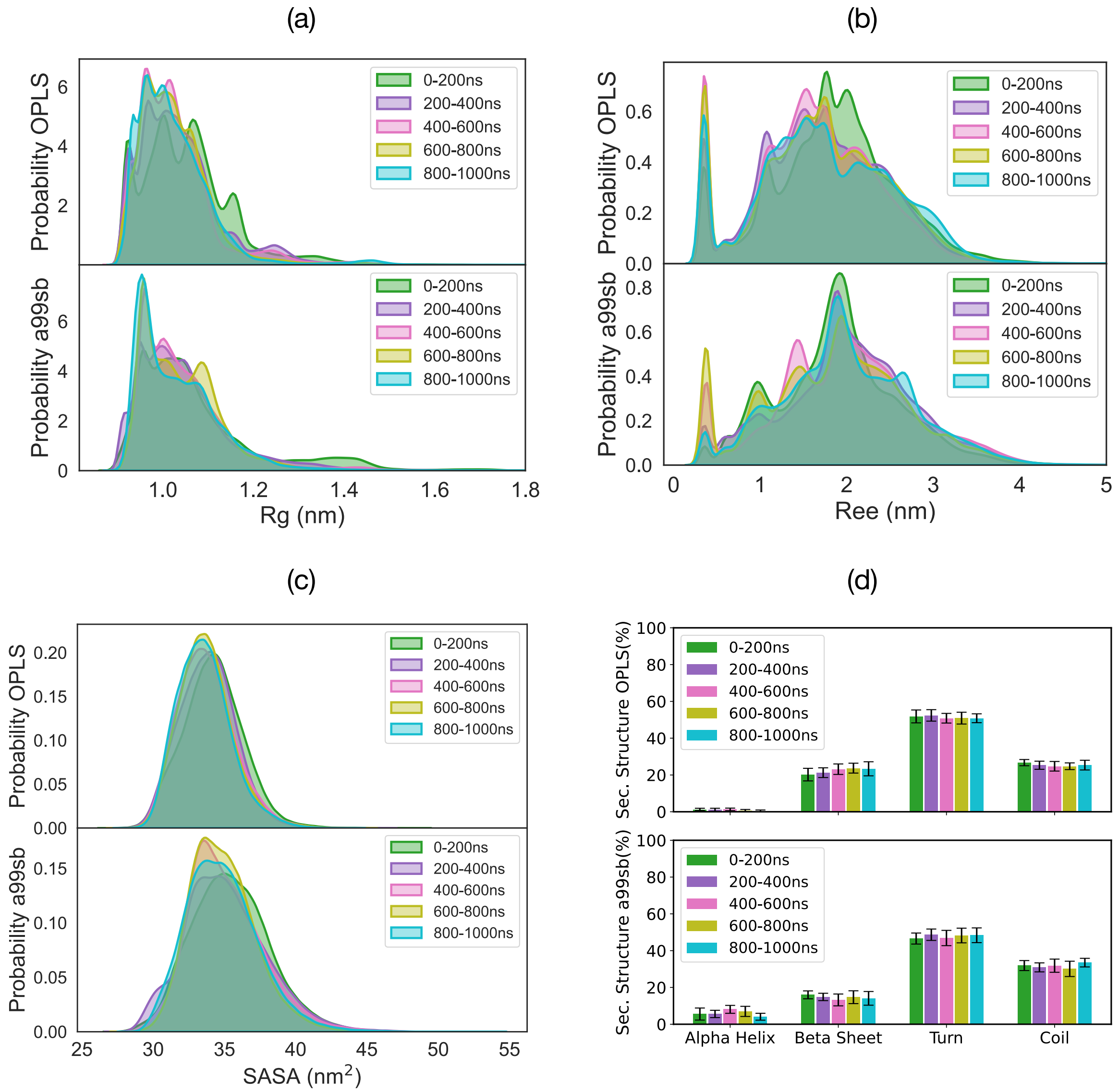

Figure S3: Time evolution of standard structural descriptors (R_g, R_{ee}, SASA, and secondary structure) was evaluated by discretizing the entire simulation into short blocks and averaging the descriptors within each block. All data refer to the first 10 replicas of T-REMD performed with the OPLS and a99SB-UCB force fields. (a) Radius of gyration. (b) End-to-end distance. (c) Solvent-accessible surface area (SASA). (d) Secondary structure (α-helix, β-Sheet, Turn and Coil).

Overall, the distribution profiles obtained from different time blocks closely overlap from 400 ns onward, demonstrating consistent sampling across the simulation and supporting its convergence.

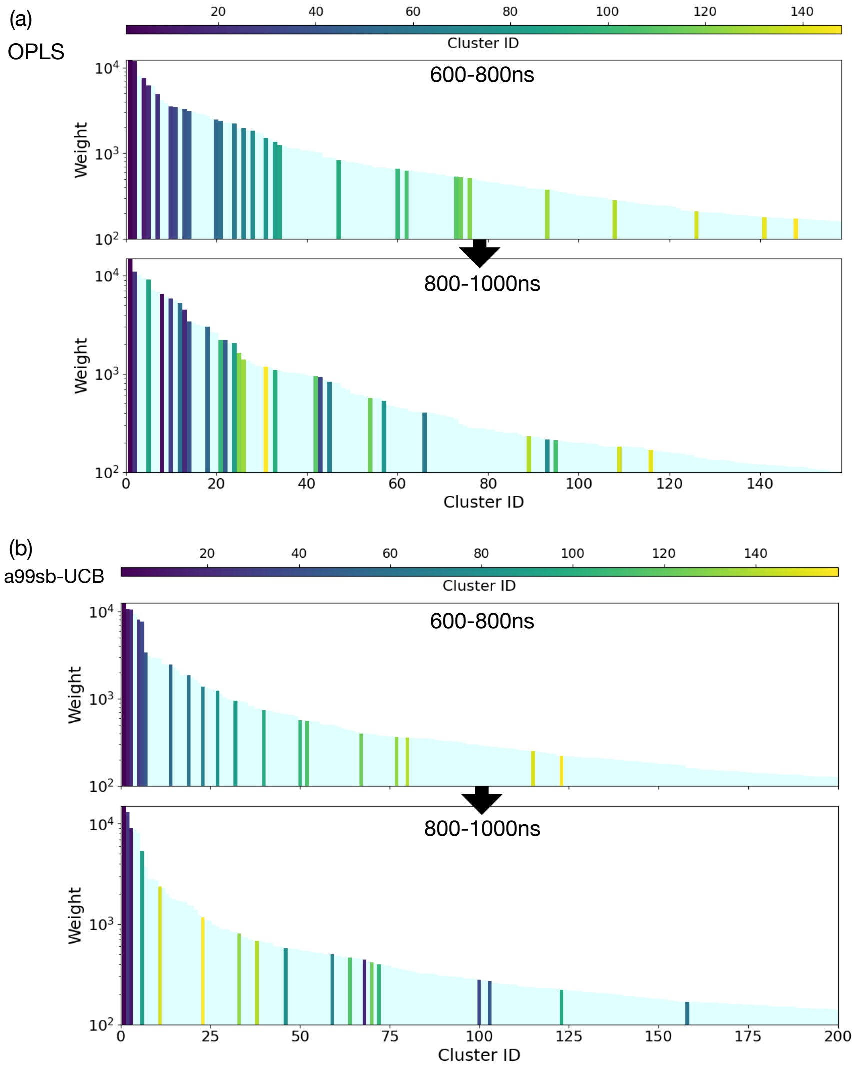

Figure S4: Convergence was evaluated by analyzing the evolution of clusters over time. Clustering was performed in 200 ns intervals for both force fields using the GROMOS algorithm[1] in GROMACS[2], considering only Cα atoms and a cutoff of 0.28 nm. Clusters with more than 100 structures were compared across consecutive intervals by taking the central structure of each cluster and calculating its RMSD against all central structures from the subsequent interval. Bar colors follow the colormap and encode cluster rank by population in the 600–800 ns interval (dark purple = most populated, yellow = least populated); the same color is assigned to a cluster in the subsequent interval if its central structure has $RMSD < 0.28$ nm against any central structure from the prior interval, enabling visual tracking of cluster identity across time windows. The light blue shaded area represents the smoothed weight envelope of the full distribution and serves as a visual reference only. We observe that clusters in the tail (rare conformations) of one interval can become dominant in the next, indicating that the system explores a broad conformational space and transitions between different metastable states.

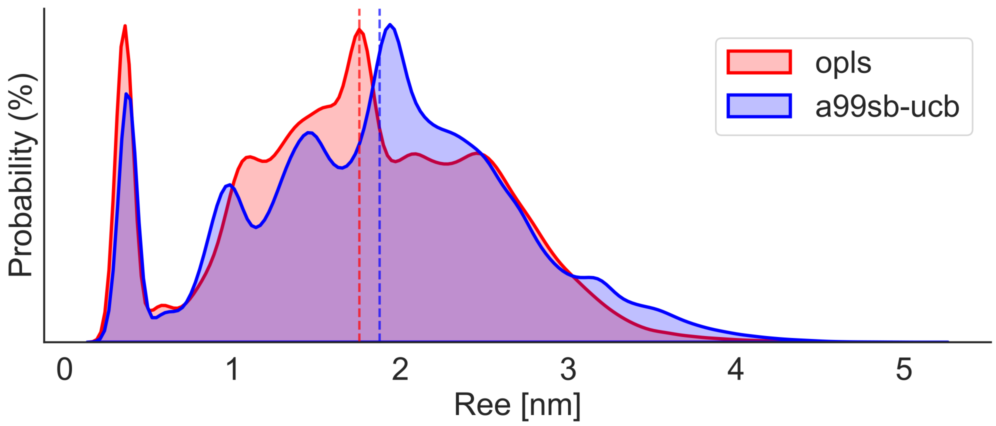

Figure S5: Comparison of the distribution of R_{ee} for the two different force fields. The average R_{ee} values of distributions ($\sim$1.76 nm and $\sim$1.88 nm for OPLS and a99SB-UCB, respectively) are consistent with previous simulations[3], but lower than the value obtained from FRET experiments[4] (see Table 1 main text). The discrepancy between the simulated and experimental data reported in Ref.[4] may arise from methodological limitations, most likely due to the presence of FRET labels and the specific orientation of the donor and acceptor dyes. Furthermore, it is noteworthy that the reference MD simulations used to validate the experimental data in that study were conducted in a simulation box of insufficient size, which may have adversely affected intra-peptide interactions, as delineated in[5] and in[6].

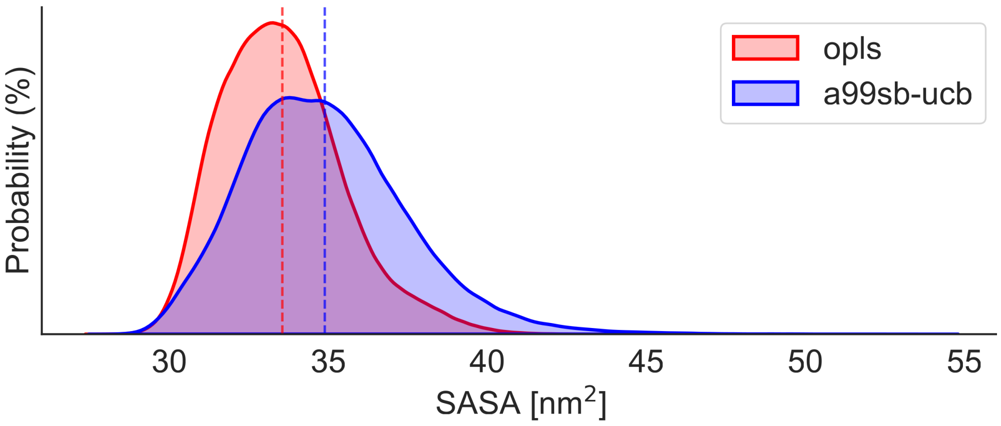

Figure S6: Comparison of the SASA distribution profiles for the two force fields shows that they align closely. However, the a99SB-UCB distribution reveals that the peptide is in general more exposed to the solvent due to its tendency to adopt elongated and less structured conformations compared to OPLS, as further supported by the analysis of R_g, R_{ee} and the secondary structure.

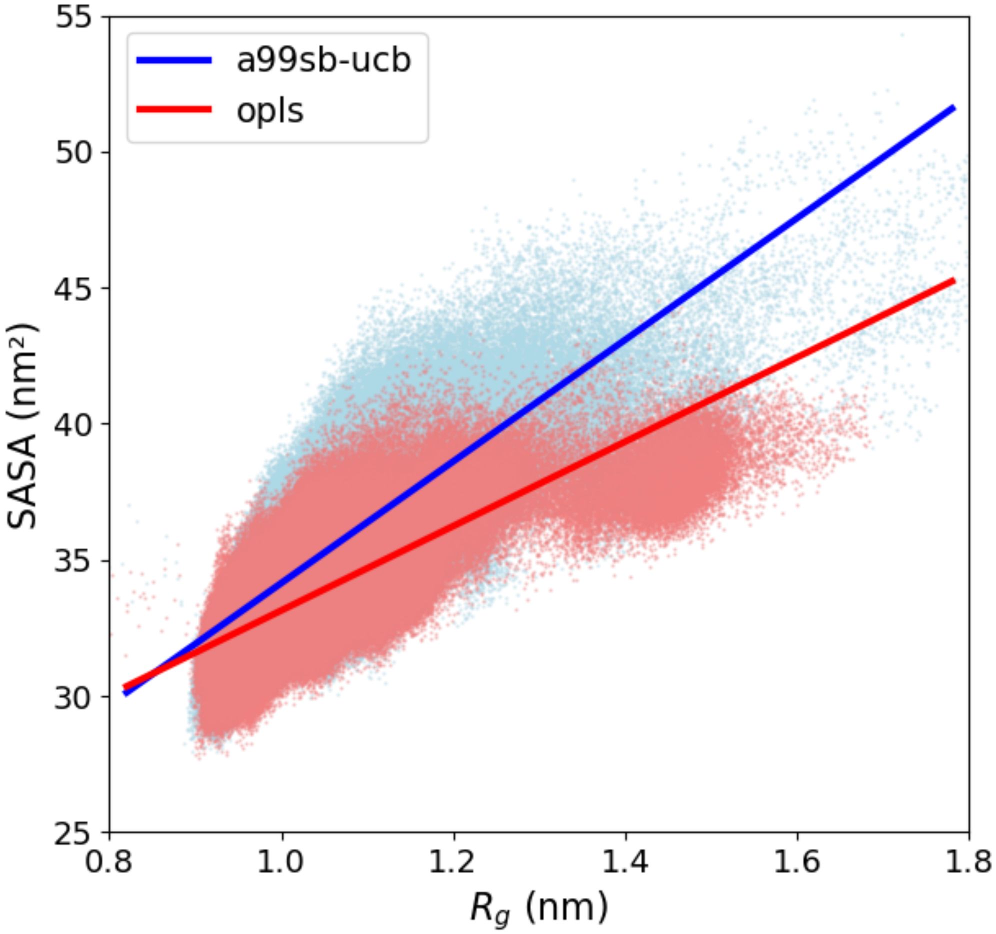

Figure S7: Correlation plot between R_g and SASA, revealing relationships and trends across both datasets. Comparison between the force fields further highlights the tendency of a99SB-UCB to favor peptide conformations that are more solvent-exposed, less structured, and biased toward elongated states compared to OPLS.

Table S1: Comparison of J-coupling values for residues 17–21 between experimental data and values obtained using OPLS, a99SB-UCB, and a merged dataset combining OPLS and a99SB-UCB results.

Rresidue	Exp. value	OPLS	a99sb-UCB	OPLS+a99sb-UCB
L17	6.63 ± 0.15	7.37 ± 0.01	6.09 ± 0.03	6.73 ± 0.02
V18	8.20 ± 0.12	7.94 ± 0.05	7.36 ± 0.04	7.65 ± 0.02
F19	7.72 ± 0.10	7.56 ± 0.01	6.92 ± 0.02	7.24 ± 0.02
F20	7.67 ± 0.11	8.05 ± 0.02	7.33 ± 0.02	7.69 ± 0.02
A21	5.56 ± 0.10	7.54 ± 0.02	7.31 ± 0.02	7.42 ± 0.02

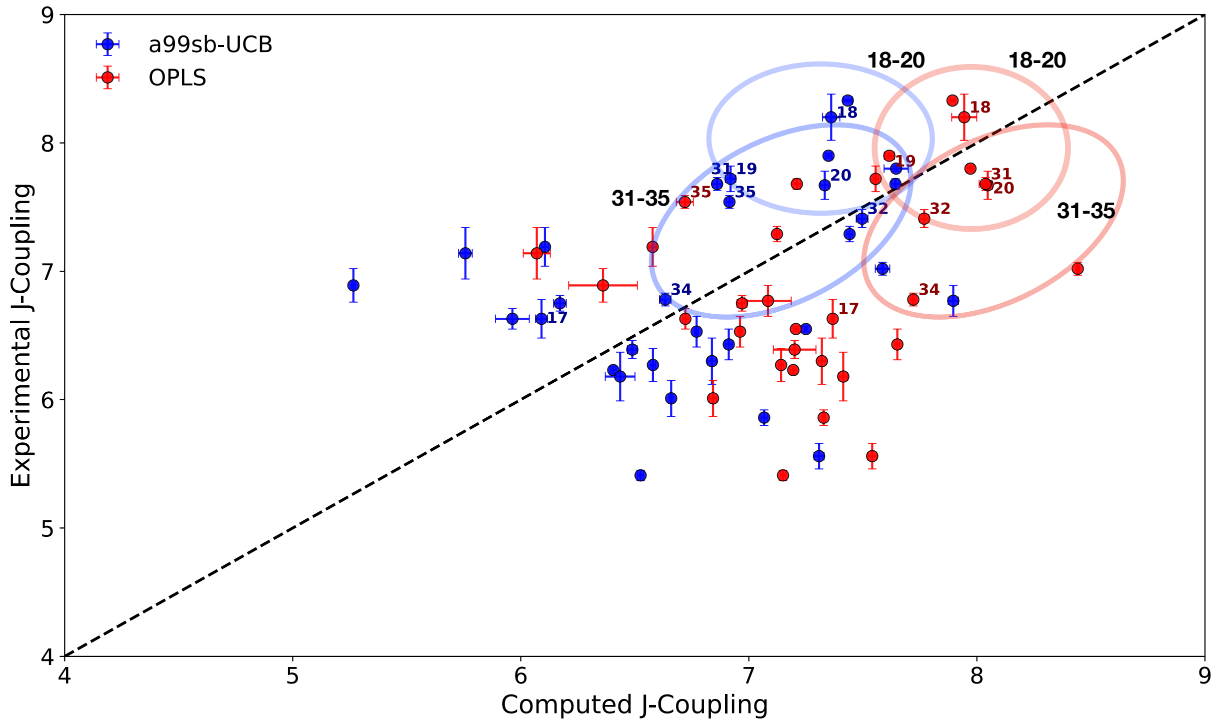

Figure S8: Comparison of experimental[7] and computed $^3J_{HN-H\alpha}$ couplings for individual Aβ42 residues using a99SB-UCB (blue) and OPLS (red) force fields. Error bars indicate standard errors. The dashed line represents the ideal correlation. A visual inspection indicates that $^3J_{HN-H\alpha}$ couplings obtained with a99SB-UCB tend to be lower than those from OPLS, consistent with the higher β-sheet content predicted by OPLS. Residue numbers corresponding to specific regions of the sequence are indicated, with the regions spanning residues 18–20 and 31–35 highlighted for both force fields. For residues 18–20, J-couplings computed with OPLS show very good agreement with experimental data, lying close to the diagonal, while a99SB-UCB slightly underestimates them, placing points above the diagonal. In the 31–35 region, a99SB-UCB slightly underestimates J-couplings, although residues 32–34 show good agreement with experiments (near in the diagonal). In contrast, OPLS tends to overestimate the J-couplings in this segment.

Table S2: Pearson coefficients between HS and each descriptor (percentage of β-sheet, α-helix, turn, coil, R_g and SASA).

	β-sheet	α-helix	turn	coil	R_g	SASA
r (600-800 ns), a99SB-UCB	-0.24	0.04	0.22	-0.12	-0.67	-0.64
r (600-800 ns), OPLS	-0.28	-0.13	0.14	0.10	-0.65	-0.60
r (800-1000 ns), a99SB-UCB	-0.22	0.02	0.19	-0.05	-0.55	-0.24
r (800-1000 ns), OPLS	-0.38	-0.01	0.21	0.18	-0.59	-0.47

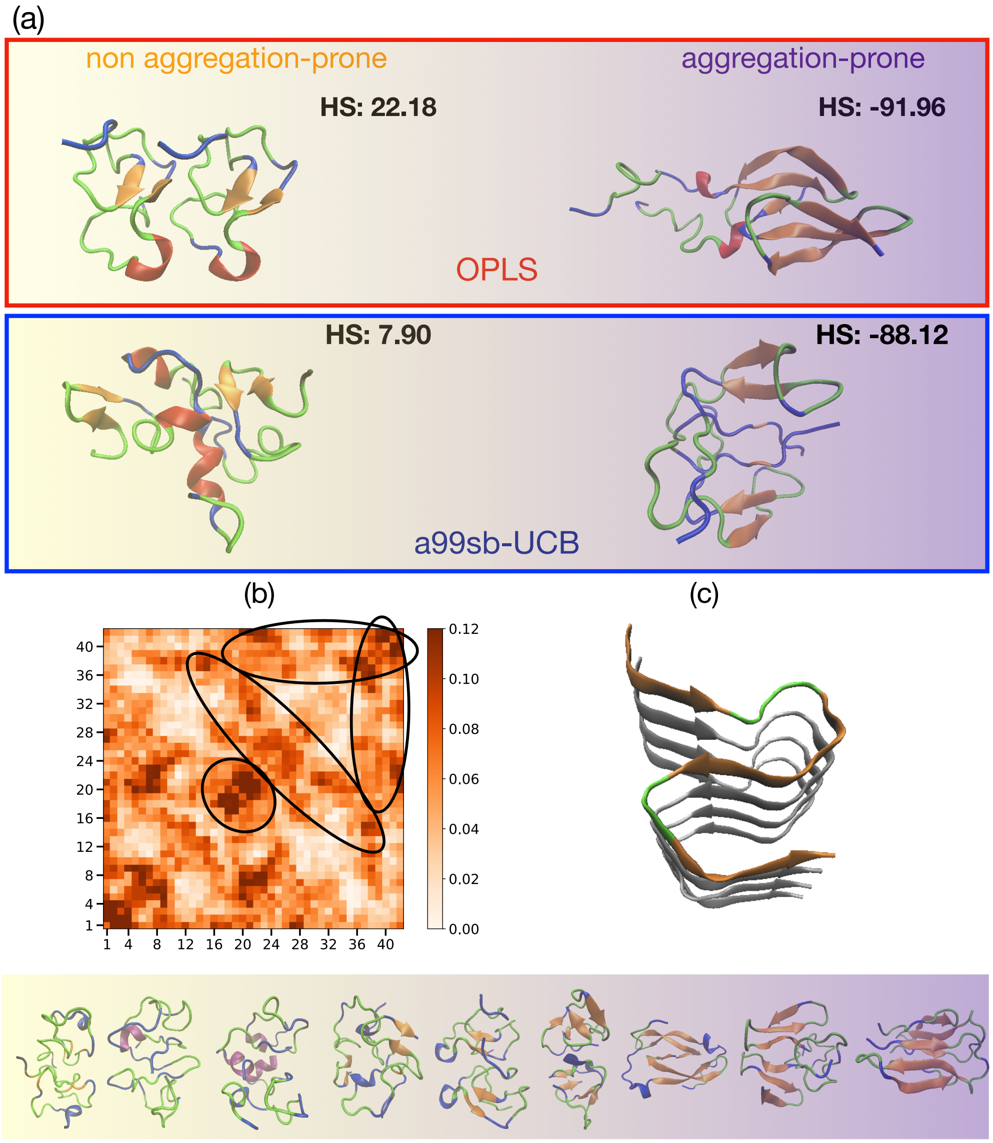

Figure S9: (a) Dimers obtained using HADDOCK docking, with the highest HADDOCK Score (HS), indicating lower aggregation propensity, and with the lowest HS, indicating higher aggregation propensity. (b) Interaction probability matrix between two monomers, computed from all docking complexes obtained by docking each structure with itself. The matrix represents the probability of interaction between residues across all sampled complexes. Selected complexes are shown, corresponding to non-aggregation-prone and aggregation-prone conformations. (c) Fibril structure (PDB ID: 2NAO).

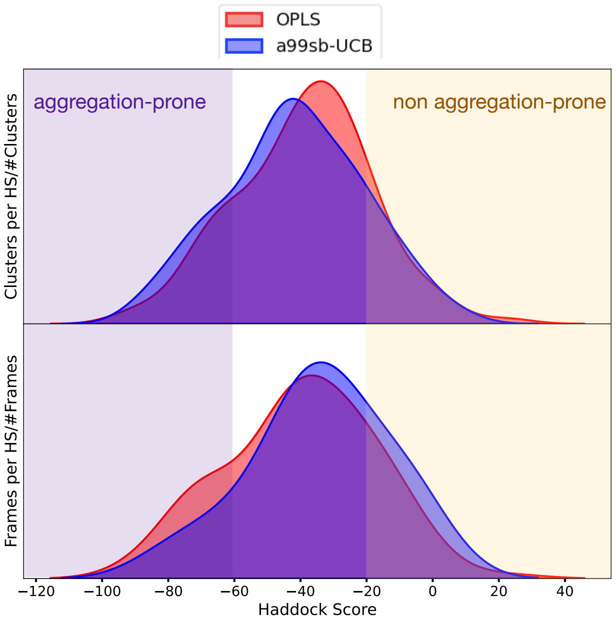

Figure S10: (a) Distribution of HS values for representative conformations: unweighted (top), and weighted by cluster populations (bottom). The top panel shows the distribution of HS values for single representative conformations of each cluster, reflecting the distribution of HS per cluster. In contrast, the bottom panel reports the same distribution after weighting each cluster by its population, reflecting the general HS distribution in spite of the size of the formed clusters. In the upper panel, a99SB-UCB produces a larger number of aggregation-prone clusters than OPLS, while the general distributions shows a shift of OPLS to lower (more aggregating) HS values. This indicates that OPLS generates fewer aggregation-prone conformations that are longer-lived, thereby enriching the cluster population. In contrast, a99SB-UCB samples more such conformations but with shorter lifetimes, smoothing the distribution. Thus, a99SB-UCB better captures the dynamic nature of Aβ, whereas OPLS tends to produce a more discretized cluster distribution, with fewer but more highly populated states.

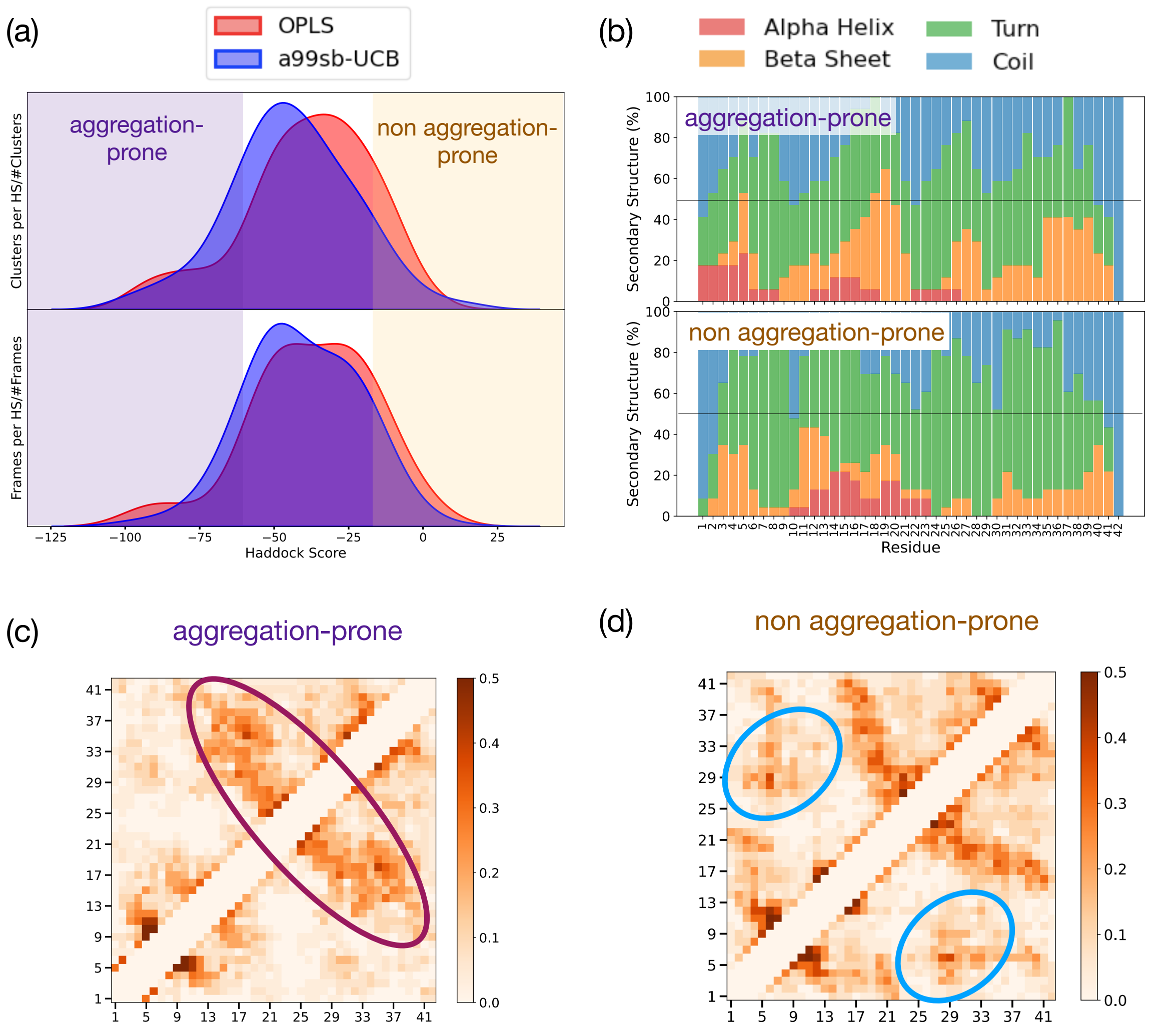

Figure S11: Analysis of the block 600-800ns (a) Haddock score distirbution obtained considering all clusters (top), or weightened clusters (bottom). (b) Secondary structure distribution per residue, obtained from structures with high aggregation propensity (top,HADDOCK score < -60) and low aggregation propensity (bottom, HADDOCK score > -20). (c) Intrapeptide interaction matrix for aggregation-prone conformations. High contact probability regions obtained are shown in purple or cyan, corresponding to the structures shown in Figure 4c. (d) Same as (c) but for non aggregation-prone conformations.

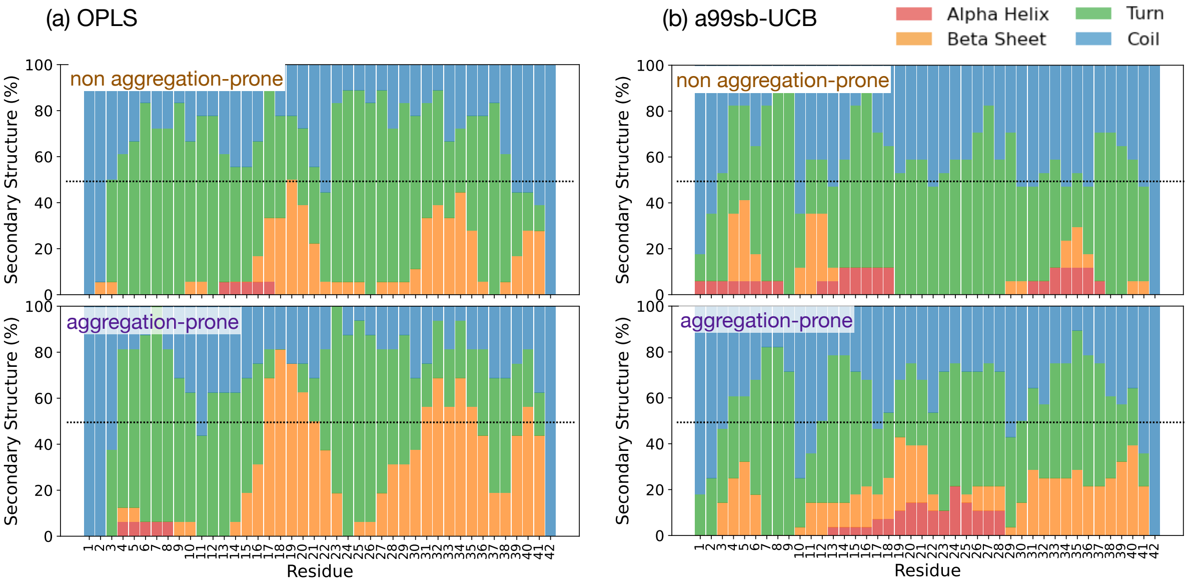

Figure S12: Secondary structure distribution per residue, obtained from structure with low aggregation propensity (top) and high aggregation propensity (bottom) for OPLS (a) and a99sb-UCB (b). OPLS promotes aggregation through a β-hairpin structure, whose content is enhanced in aggregation-prone conformations compared to non-aggregation-prone ones. A similar trend is observed for a99SB-UCB, where aggregation-prone conformations also show increased β-sheet content in these regions; however, they are additionally characterized by the presence of disordered regions, particularly around residues 10–12 and 29–30. Interestingly, when comparing the aggregation-prone conformations of a99SB-UCB with the non-aggregation-prone conformations of OPLS, the overall β-sheet content appears to be similar. This suggests that β-sheet content alone may not be sufficient to drive aggregation. Instead, in the case of a99SB-UCB, the coexistence of β-structures with disordered regions seems to play a crucial role in promoting aggregation.

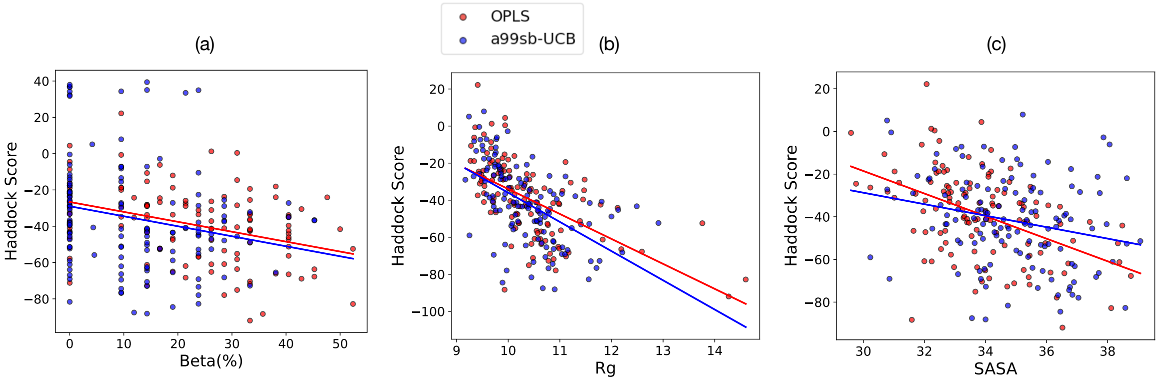

Figure S13: Analysis of the final block 800-1000 ns. (a) Correlation plots between HS and percentage of β-sheet in Aβ, (b) between HS and radius of gyration (R_g) and (c) between HS and SASA.

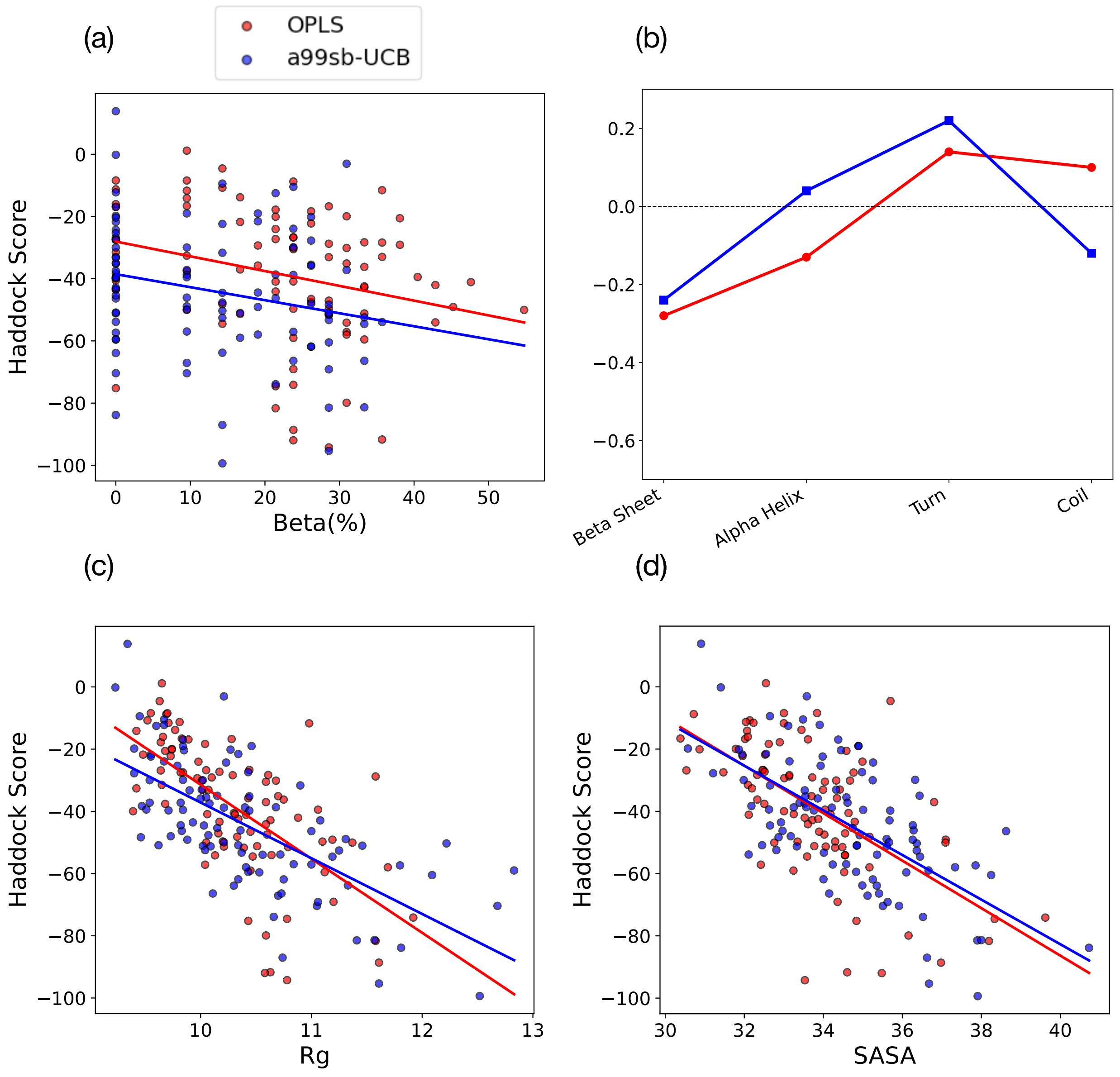

Figure S14: Analysis of the block 600-800 ns (a) Correlation plots between HS and percentage of β-sheet in Aβ, (b) pearson coefficients between HS and secondary structure elements, (c) correlation plots between HS and radius of gyration, (d) correlation between HS and SASA.

Ensemble Validation Protocols

To validate the simulated ensembles, structural and NMR observables were computed and compared with experimental data. Specifically, the radius of gyration (R_g), end-to-end distance (R_{ee}), chemical shifts, and J coupling constants were analyzed for each ensemble generated with the OPLS and a99SB-UCB force fields. The following sections report all the details, parameters and the relations used for the evaluation of each observable.

End-to-end distance and FRET scaling

The apparent end-to-end distance derived from FRET efficiency was estimated according to

$$E_{\text{FRET}} = \frac{1}{1 + (R'_{ee}/R_0)^6} \tag{1}$$

where R_0 is the Förster radius (∼5.2 nm for the Alexa 488/647 dye pair). The simulated R_{ee} values were scaled to account for the linker and dye contribution by treating them as 12 additional residues and assuming Gaussian chain scaling:

$$R'_{ee}(t) = R_{ee}(t) \left(\frac{N + 12}{N} \right)^{0.5} \tag{2}$$

where $N = 42$ is the number of residues in Aβ42. Considering $E_{\text{FRET}} = 0.6 \pm 0.1$, the estimated $R'_{ee} = 4.85$ nm corresponds to $R_{ee} = 4.28$ nm.

Chemical shifts and J couplings

Chemical shifts for the Cα atoms were predicted using the SPARTA+ program[8]. J coupling constants were estimated using the Karplus equation:

$$J(\phi) = A \cos^2 \phi + B \cos \phi + C \tag{3}$$

where ϕ is the H–N–Cα–H dihedral angle, and the coefficients are $A = 7.97$, $B = -1.26$, and $C = 0.63$[9]. Uncertainties were estimated via the block-averaging method.

Comparison with experimental data

Simulated and experimental coupling constants[7] were compared using the χ^2 metric[10]:

$$\chi^2 = \frac{1}{N}\sum_{i=1}^{N}\frac{(J_{i,\mathrm{sim}} - J_{i,\mathrm{exp}})^2}{\Delta^2} \tag{4}$$

with $\Delta = 0.42$ Hz.

Analyzes were performed independently for the OPLS and a99SB-UCB ensembles, and the results were compared to assess which force field better reproduced the experimental observables. In addition, a combined ensemble was evaluated—obtained by merging conformations from both force fields— to determine whether it improved agreement with the experimental data.

References

(1) Daura, X.; Gademann, K.; Jaun, B.; Seebach, D.; Van Gunsteren, W. F.; Mark, A. E. Peptide folding: when simulation meets experiment. *Angewandte Chemie International Edition* **1999**, *38*, 236–240.

(2) Abraham, M. J.; Murtola, T.; Schulz, R.; Páll, S.; Smith, J. C.; Hess, B.; Lindahl, E. GROMACS: High performance molecular simulations through multi-level parallelism from laptops to supercomputers. *SoftwareX* **2015**, *1*, 19–25.

(3) Barz, B.; Buell, A. K.; Nath, S. Compact fibril-like structure of amyloid β-peptide (1–42) monomers. *Chemical Communications* **2021**, *57*, 947–950.

(4) Meng, F.; Bellaiche, M. M.; Kim, J. Y.; Zerze, G. H.; Best, R. B.; Chung, H. S. Highly Disordered Amyloid-β Monomer Probed by Single-Molecule FRET and MD Simulation. *Biophysical Journal* **2018**, *114*, 870–884.

(5) Weber, O.; Uversky, V. How accurate are your simulations? Effects of confined aqueous volume and AMBER FF99SB and CHARMM22/CMAP force field parameters on structural ensembles of intrinsically disordered proteins: Amyloid-β42 in water. *Intrinsically Disordered Proteins* **2017**, *5*, e1377813.

(6) Bellucci, L.; Bussi, G.; Di Felice, R.; Corni, S. Fibrillation-prone conformations of the amyloid-β-42 peptide at the gold/water interface. *Nanoscale* **2017**, *9*, 2279–2290.

(7) Roche, J.; Shen, Y.; Lee, J. H.; Ying, J.; Bax, A. Monomeric Aβ^{1-40} and Aβ^{1-42} Peptides in Solution Adopt Very Similar Ramachandran Map Distributions That Closely Resemble Random Coil. *Biochemistry* **2016**, *55*, 762–775.

(8) Shen, Y.; Bax, A. SPARTA+: a modest improvement in empirical NMR chemical shift prediction by means of an artificial neural network. *Journal of Biomolecular NMR* **2010**, *48*, 13–22.

(9) Grishaev, A.; Bax, A. An empirical backbone-backbone hydrogen-bonding potential in proteins and its applications to NMR structure refinement and validation. *Journal of the American Chemical Society* **2004**, *126*, 7281–7292.

(10) Paul, A.; Samantray, S.; Anteghini, M.; Khaled, M.; Strodel, B. Thermodynamics and Kinetics of the Amyloid-β Peptide Revealed by Markov State Models Based on MD Data in Agreement with Experiment. *Chemical Science* **2021**, *12*, 6652–6669.
